# The Effects of Cognitive Fatigue and Articulatory Suppression on Statistical Language Learning Depend on the Strength of Cognitive Resources

**DOI:** 10.1111/cogs.70178

**Published:** 2026-02-23

**Authors:** Eleonore H. M. Smalle, Evgenia Karantinou, Riikka Möttönen

**Affiliations:** ^1^ Department of Developmental Psychology Tilburg University; ^2^ Department of Experimental Psychology Ghent University; ^3^ Department of Digital Humanities University of Helsinki

**Keywords:** Statistical language learning, Cognitive fatigue, Articulatory suppression, Executive functions

## Abstract

In adults, cognitive fatigue enhances statistical language learning—the ability to detect repeating hidden patterns in continuous speech, and a core component of implicit language acquisition. This supports the cognitive cost hypothesis, which proposes that the adult cognitive architecture, especially executive functions (EFs), constrains effortless language learning. In contrast, articulatory suppression impairs statistical language learning, suggesting the involvement of lower‐level auditory‐motor mechanisms in acquiring new linguistic knowledge from speech streams. This study examined whether the effects of cognitive fatigue and articulatory suppression on statistical language learning depend on individuals’ cognitive resources (CRs). Specifically, we tested whether individual differences in late‐developing EFs (1) are associated with statistical language learning ability and (2) modulate the effects of cognitive fatigue and articulatory suppression. Fifty Dutch‐speaking first‐year university students participated in a multisession statistical‐learning experiment. EFs were assessed using three tasks measuring working memory, inhibitory control, and cognitive flexibility. Cognitive fatigue was induced using a time‐loaded dual N‐back task at participants’ maximum speed prior to speech exposure. Articulatory suppression was induced by instructing participants to whisper continuously while listening to the speech stream. Learning was assessed using a post‐exposure two‐alternative forced‐choice recognition task, where participants selected between a pseudoword from the stream and a foil. Based on a factor and cluster analysis of EF scores, participants were grouped into high and low CR groups. In line with the cognitive cost hypothesis, the high CR group performed less well in statistical language learning than the low CR group but benefited from cognitive fatigue. Articulatory suppression impaired statistical learning in the low CR group only, suggesting they rely more on auditory‐motor mechanisms. These findings demonstrate that cognitive functioning impacts statistical language learning, suggesting that the ability to acquire new linguistic knowledge from speech streams depends on the interaction between higher‐level cognitive and lower‐level auditory‐motor mechanisms.

## 1 Introduction

Statistical learning—the ability to extract probabilistic regularities from the environment—has been the focus of extensive investigation in cognitive science over the past 25 years. Today, it is widely regarded as a fundamental mechanism in almost all theories of information processing, including language acquisition (Frost, Armstrong, & Christiansen, 2019). Language learning is an essential aspect of human cognition, enabling us to build and refine internal models of the world that help us predict, interpret, and adapt to the communicative demands of our dynamic social environment. Here, we focus on variation of language acquisition across development and individuals, and especially aim to contribute to understanding the potential sources of variance in statistical language learning in adults.

From an early age, children acquire complex language skills without formal teaching (Kuhl, 2004). These skills involve identifying speech sounds and their combinations, grouping them into lexical categories, and extracting word meanings and grammatical rules. This achievement is particularly striking given that their cognitive capacities are still developing, underscoring the remarkable ability of the human brain to acquire complex skills without the need for conscious effort or extensive feedback. A central mechanism underlying this process is *statistical learning* (Aslin, 2017), the ability to detect temporal and spatial patterns in the environment simply through repetitive exposure. Statistical learning was first demonstrated in the domain of spoken language acquisition by Saffran, Aslin, and Newport (1996), who showed that 8‐month‐old infants track transitional probabilities (TPs) between spoken syllables to discover word boundaries in speech (Aslin, Saffran, & Newport, 1998; Saffran et al., 1996). TPs of speech sounds are higher within word‐forms than between, providing a reliable cue for word segmentation when listening to unfamiliar speech—one of the first challenges in spoken language acquisition (Kuhl, 2004). In a similar vein, Marcus, Vijayan, Bandi Rao, and Vishton (1999) demonstrated that 7‐month‐old infants can learn nonadjacent dependencies between sounds (“abstract rules”) from continuous speech sounds and generalize these rules to new structures (Marcus et al., 1999). This latter is a key feature of grammatical processing and language acquisition more broadly—highlighting the brain's early ability to generalize from limited exposure to a rich variety of linguistic forms.

After these seminal findings on early language development, work on statistical learning exploded (Frost et al., 2019). Accumulating research showed that the ability to detect predictive patterns based on probabilistic information—such as adjacent and nonadjacent TPs—extends well beyond language domains and infants. The capacity is not only evident in various other cognitive functions (e.g., Frost et al., 2019), but also in older learners including children and adults (Palmer, Hutson, & Mattys, 2018; Raviv & Arnon, 2018; Saffran, Newport, Aslin, Tunick, & Barrueco, 1997), and even in newborns (Fló et al., 2019; Teinonen, Fellman, Näätänen, Alku, & Huotilainen, 2009). Today, statistical learning has become a cornerstone theory in cognitive science and is widely accepted as a powerful learning mechanism underlying various stages of language acquisition, in both first and second language learning, across development (Thiessen, Girard, & Erickson, 2016).

Statistical language learning abilities persist throughout life, but the efficiency with which we acquire language is characterized by substantial variation (Bates, Dale, & Thal, 1996). While language development evolves mostly effortlessly in childhood, learning becomes more challenging in adulthood (Chen & Hartshorne, 2021; Johnson & Newport, 1989; Lenneberg, 1967), with substantial individual differences in both groups (Kidd, Donnelly, & Christiansen, 2018). As early as 2005, the question of why critical periods exist in language learning attracted major scientific attention (Kennedy & Norman, 2005). Today, the existence and shape of individual differences in language learning, including a critical period, remain debated (Kidd et al., 2018; Zwart, Vissers, Kessels, & Maes, 2019). Some researchers argue for a discontinuity in learning outcomes across age (e.g., Newport, 1990; Hartshorne et al., 2018), whereas others suggest a more continuous decline without sharp boundaries (Hakuta et al., 2003). Moreover, multiple factors are likely to contribute to children's advantages in language acquisition, some of which may also account for broader individual differences—such as superior neural plasticity, longer cumulative exposure, social motivations to conform to peers, and/or reduced interference from a well‐learned first language (Birdsong, 2018). Regarding the latter, specific evidence in the field of statistical learning theory suggests that adults bring in a wealth of prior linguistic knowledge, which can facilitate the learning of regularities that align with their native language (e.g., Smalle & Szmalec, 2022), but can also interfere when novel patterns conflict with entrenched structures (Elazar et al., 2022; Siegelman, Bogaerts, Elazar, Arciuli, & Frost, 2018). For instance, first language knowledge can interfere with word segmentation, leading learners to rely on familiar cues at the expense of detecting new statistical regularities (Finn & Hudson Kam, 2008). Similar L1‐driven biases have been observed in learning more complex structures, such as the acquisition of nonadjacent vocalic dependencies, where Khalkha Mongolian speakers exhibited preferences shaped by their native vowel system (LaCross, 2015). Together, these findings highlight that, although the capacity for statistical learning persists across the lifespan, its role in language acquisition is fundamentally shaped by the underlying cognitive and linguistic resources of the individual learners. As a result, important differences do not only emerge between children's and adults’ learning trajectories, but also among individual learners more broadly.

Here, we focus on one influential proposal that accounts for variation in language learning—the less‐is‐more hypothesis (Newport, 1990). Although experimental evidence was limited for many years, the hypothesis has recently regained momentum in the field of statistical learning (Smalle & Möttönen, 2023). In 1990, Elissa Newport suggested that children may have a unique advantage in learning early phonological and grammatical aspects of languages because their limitations in higher cognitive functions—such as short‐term memory and attention—force them to focus on, and analyze, finer‐grained aspects of complex linguistic input. This constraint, paradoxically, may help them acquire language more effectively. Higher‐order cognitive functions are typically referred to as executive functions (EFs) and include late‐developing cognitive abilities such as working memory, inhibitory control, cognitive flexibility, planning, reasoning, and problem solving (Diamond, 2013). They follow a strong developmental trend, linked to prefrontal cortex maturation (e.g., Crone, 2009; Paz‐Alonso, Bunge, Ghetti, & Board, 2014), with a rapid development in late childhood to mid‐adolescence (10–15 years old) before they stabilize in the early adulthoods (Best & Miller, 2010; Ferguson, Brunsdon, & Bradford, 2021; Poon, 2018; Tervo‐Clemmens et al., 2023). There is now accumulating evidence that the development of EFs constrains statistical learning processes underlying early stages of language acquisition, in line with Newport's original less‐is‐more hypothesis (Smalle & Möttönen, 2023). For instance, research in adults has shown that disrupting the late‐developing cognitive‐control system in the prefrontal cortex via noninvasive brain stimulation and via inducing cognitive fatigue boosts word segmentation in speech (Smalle, Daikoku, Szmalec, Duyck, & Möttönen, 2022). These cognitive depletions particularly enhance adults’ implicit memory for the newly acquired words, in a similar way as children do (Smalle & Bogaerts, 2024).

The suggestion that the adult cognitive architecture constrains underlying statistical learning processes, and can partly explain the increase of challenges in early stages of language learning from childhood to adulthood, is referred to as the *cognitive cost* hypothesis (Smalle & Möttönen, 2023). The hypothesis is closely related to a broader competition framework in statistical learning, mainly investigated in the perceptual‐motor field, that shows a competitive interaction between explicit, prefrontal‐mediated executive processes and domain‐general implicit statistical learning mechanisms (e.g., Ambrus et al., 2020; Park, Janacsek, Nemeth, & Jeon, 2022; Tóth et al., 2017). Dual‐process models of human learning capture this interaction by proposing that statistical learning is largely supported by implicit, model‐free processes, which in certain situations compete with explicit, model‐based processes (Beierholm, Anen, Quartz, & Bossaerts, 2011; Daw, Niv, & Dayan, 2005; Poldrack & Packard, 2003). This interaction is believed to be controlled by the prefrontal cortex: when a flexible, but cognitively costly strategy (i.e., model‐based learning) is favored in a task, the prefrontal cortex suppresses the brain's default model‐free processes that characterize statistical learning (Lee, Shimojo, & O'Doherty, 2014). Developmental and neural evidence suggests that the competition becomes stronger as the prefrontal cortex matures, providing a direct explanation for why adults often struggle in learning new skills implicitly (Janacsek, Fiser, & Nemeth, 2012; Tóth‐Faber et al., 2024). Interestingly, research in this field has also begun to reveal competitive relationships on an interindividual level, specifically between adults’ cognitive resources (CRs) and their statistical learning abilities. For instance, Virag et al. (2015) found a negative correlation between a composite EF score (including scores from a digit span task, listening span task, counting span task, and letter fluency task) and individual performance on the Alternating Serial Reaction Time task (ASRT task), which is a task commonly used to measure visuo‐motor statistical learning; lower EFs were associated with better statistical learning (Virag et al., 2015). More recently, Pedraza et al. (2024) investigated the relationship between statistical learning and EFs across two large university student samples (age under 35), by using the ASRT task and a battery of neuropsychological tests (Pedraza et al., 2024). Results revealed a consistent, modest negative correlation that was particularly driven by EF components related to verbal fluency and working memory, suggesting a potential competitive interaction between implicit learning and adults’ available CRs. A similar competitive relationship may be found in the language domain, though there is little contrasting evidence for this idea (e.g., Kalra, Gabrieli, & Finn, 2019) with some studies finding no correlation (e.g., Siegelman & Frost, 2015) or a positive correlation between statistical learning and working memory (e.g., Arnon, 2020; Misyak & Christiansen, 2012). Hence, more research is needed to investigate this relationship further.

While much of the existing work on statistical language learning has emphasized underlying domain‐general cognitive mechanisms, another growing body of research highlights the importance of modality‐specific systems in this process. In 2015, Frost and colleagues proposed a theoretical framework to explain how statistical learning can operate across cognitive domains and modalities while still demonstrating specificity. They argued that statistical learning involves domain‐general computational principles, but that these are implemented by distinct neural networks depending on the properties of the input signal (e.g., visual, auditory, or somatosensory). In this view, differences in statistical learning across modalities are attributable to modality‐specific encoding constraints—for instance, the auditory cortex is highly sensitive to temporal information but less tuned to spatial information. This perspective of domain‐general computations shaped by modality‐specific constraints is also covered in more recent work by Conway (2020), who proposed that dynamic interactions between modality‐specific brain regions and higher‐order control areas, such as the prefrontal cortex, likely underlie statistical learning. This view has been further extended by Bogaerts et al. (2022), who critically examine the notion of a single, domain‐general statistical learning capacity and argue that individual differences in statistical learning are better understood as emerging from task‐, modaity‐, and context‐specific mechanisms rather than a unitary learner trait. Within the language domain, a recent active line of research has provided evidence that auditory−motor interaction mechanisms support individual differences in statistical language learning (Assaneo et al., 2019; Boeve, Möttönen, & Smalle, 2024; Orpella et al., 2022). Assaneo et al. (2019) discovered that people who spontaneously synchronize their speech motor movements with an isochronous speech signal, that is, high synchronizers, are better statistical language learners than low synchronizers, at least with respect to word segmentation. Moreover, Orpella et al. (2022) demonstrated that statistical learning in high synchronizers is impaired by articulatory suppression (i.e., whispering a repetitive sound) during the speech exposure. This finding was recently replicated by Boeve et al. (2024), who additionally showed that this effect is specific to the language domain. Together, these findings suggest that in contrast to constraining higher‐level cognitive mechanisms, domain‐specific low‐level auditory‐motor mechanisms play an additional *supporting* role in statistical language learning, such as word segmentation. It remains unknown whether these mechanisms interact with each other, or whether individuals with high and low CRs rely equally on such low‐level mechanisms in acquiring new linguistic knowledge. Filling this gap in knowledge would significantly expand the cognitive cost hypothesis and improve understanding of how CRs interact with supporting speech‐motor mechanisms in early stages of language acquisition.

In the current study, we aimed to find out whether differences in CRs affect statistical language learning ability, and whether these differences modulate the contributions of higher‐level cognitive mechanisms and lower‐level auditory‐motor mechanisms to this ability. Given ongoing concerns about the reliability of statistical learning measures, particularly on indirect post‐exposure measures (e.g., Kidd et al., 2020, 2023), we adopted a group‐based approach to mitigate these issues. Specifically, we examined the effects of cognitive fatigue and articulatory suppression on the outcomes of statistical language learning and tested whether the strength of these effects differ between individuals with high versus low CRs. Participants’ CRs were determined using a short battery of cognitive tasks related to verbal fluency and working memory, inspired by the study of Pedraza et al. (2024), that is, a backward and forward digit span, a response inhibition (Simon) task, and a cognitive flexibility (Switch) task. A high and low CR group was created based on a factor and cluster analysis of these task scores. Cognitive fatigue was induced prior to listening to a speech stream by using an individually tailored cognitive fatigue task (i.e., Time‐Load‐Dual‐Back task) in which participants perform a dual working‐memory task at their maximum speed capacity (Borragán, Slama, Bartolomei, & Peigneux, 2017). Articulatory suppression was induced by having participants whisper continuous sounds when listening to a speech stream, similar to earlier work (Orpella et al., 2022; Boeve et al., 2024). Statistical learning outcome was tested using the commonly used two‐alternative forced‐choice memory task (recognizing the hidden word‐forms from the structured exposure streams from foils) following the speech exposure. A memory judgment procedure was added to dissociate between implicit and explicit knowledge of the learned word‐forms. Reflection‐based measures such as the word recognition task often underestimate how much knowledge individuals acquire (Christiansen, 2019). In fact, in a series of studies, Batterink and colleagues showed via a memory judgment procedure that these tasks reveal solely explicit knowledge in adults (Batterink & Paller, 2017; Batterink, Reber, Neville, & Paller, 2015). In contrast, children's performance (and of adults under cognitive fatigue; Smalle et al., 2022) show evidence for both implicit and explicit knowledge (Moreau, Joanisse, Mulgrew, & Batterink, 2022; Smalle & Bogaerts, 2024). Given that implicit memory traces in statistical learning are more stable over time than explicit memory traces (Liu, Forest, Duncan, & Finn, 2023), a clearer dissociation of the knowledge acquired after exposure may provide important insights into age‐related changes and individual differences in efficient skill acquisition, as well as into the inconsistencies observed therein across domains (e.g., Raviv & Arnon, 2018).

Overall, in line with the cognitive cost hypothesis, we predicted that individuals with high CRs would be less skilled at statistical language learning and would benefit more from cognitive fatigue than those with low CRs. Additionally, we hypothesized that cognitive fatigue would primarily impair the acquisition of explicit knowledge, while potentially facilitating the acquisition of implicit knowledge. This latter would be consistent with a bottleneck assumption proposed by Smalle et al. (2022), suggesting that the adult brain imposes a “bottleneck” that prioritizes explicit memory mechanisms. Disrupting higher‐level EFs via cognitive fatigue abolishes this “bottleneck” and, as a consequence, improves adults’ capacity to simultaneously store linguistic knowledge in both implicit and explicit memory systems. In line with previous research highlighting the supporting role of auditory‐motor mechanisms, we predicted that participants’ ability to acquire new linguistic knowledge would be impaired by articulatory suppression during exposure to speech streams (e.g., Orpella et al., 2022; Boeve et al., 2024). No difference between high and low CR groups in the effect of articulatory suppression would suggest that they rely equally on auditory‐motor mechanisms, whereas a greater effect in the low than high CR group would suggest interactive mechanisms, such that CRs constrain the use of lower‐level auditory‐motor mechanisms in the acquisition of new linguistic knowledge from speech streams.

## 2 Method

### 2.1 Participants

Fifty first‐year psychology students from Ghent University (Belgium) participated in the study. This sample size is considered sufficient for allowing a subgroup split and providing adequate power to test a two‐way interaction in a mixed design (Brysbaert, 2019; see also, Orpella et al., 2022; Smalle et al., 2022, for similar sample sizes), see also a formal power analysis further below. All participants completed a three‐session experiment in exchange for course credits. One participant was a priori excluded from data‐analysis because of questionable task compliance. An additional three participants did not complete all three sessions and were replaced by new participants. Hence, the data from 49 participants are considered for final analysis (average age = 18.9 ± 2.5_SD_, minimum age = 17, maximum age = 30, 6 males, 4 left‐handed). All participants were highly fluent in Dutch (two as non‐native Dutch speakers), and none reported a history of developmental disorders. Written informed consent was obtained prior to the start of the experiment, and debriefing took place afterward. The research was approved by the Ethical Commission of the Faculty of Psychology and Educational Sciences at Ghent University (reference: 2021/4).

### 2.2 Tasks and procedures

**Fig. 1:**
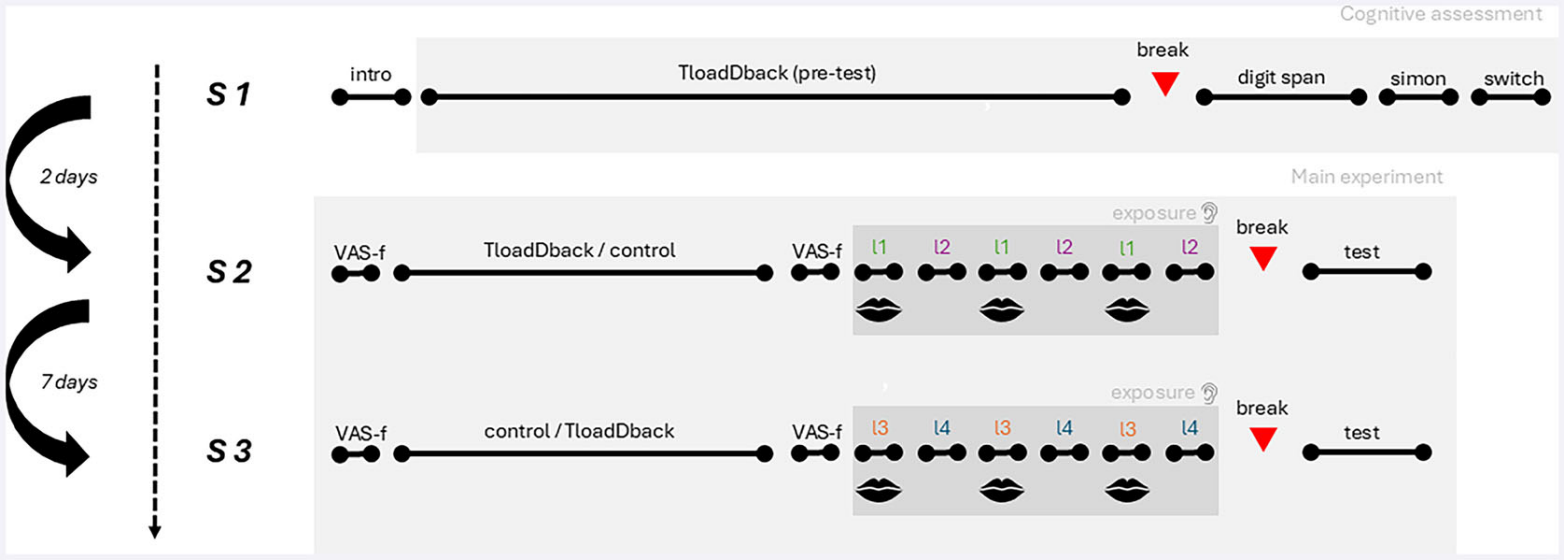
Within‐subject study procedure. Cognitive fatigue was manipulated between sessions by having the participants complete a TloadDback task under their max. processing speed capacity or an equally time‐filling control task prior to the language presentation (order across sessions counterbalanced across participants). Articulatory suppression was manipulated within session by presenting two languages under a passive listening and a whispering condition (allocation of the languages to each condition counterbalanced across participants). The manipulations are done on a within‐subject level.

The study procedure is outlined in Fig. 1. Participants attended three lab sessions, spaced 2−3 days apart for the first two sessions and 7 days apart for the second and third sessions.^1^ Each session lasted 1 h. In Session 1 (S1), participants read the informed consent, completed a background questionnaire, and underwent a cognitive assessment, including a pretest on the Time load Dual‐Back (TloadDack) task to determine participants’ maximum processing speed capacity that is also needed for inducing cognitive load in the main experiment (see further), a forward and backward digit span test, Simon task, and Switch task. In Session 2 (S2), participants were randomly assigned to one of two conditions: half (*N* = 25) performed the TloadDack task at their maximum speed capacity (inducing cognitive load and fatigue), while the other half (*N* = 24) performed a control task (solving word anagrams). Cognitive fatigue was assessed before and after the tasks using the 18‐item Visual Analogue Scale for Fatigue (VAS‐f). Participants then listened to 1‐min clips of two structured syllable streams (“languages,” l1 and l2) while either whispering the sound “tah” (articulatory suppression) or passively listening. Each language was presented three times in alternation, with the order of the conditions counterbalanced. A word recognition test followed a 5‐min break, assessing knowledge of the hidden words in both languages. In Session 3 (S3), the procedure was repeated with two novel languages (l3 and l4), and participants switched task condition (TloadDack or control) compared to Session 2. In other words, the experiment manipulated cognitive fatigue and articulatory suppression completely within subjects, with a counterbalanced order for cognitive fatigue (i.e., half of the participants in Session 2 and half of the participants in Session 3). All sessions were conducted using the Gorilla experiment builder (Anwyl‐Irvine et al., 2020) on a Windows 10 desktop computer in an isolated university lab.

#### 2.2.1 Time load dual‐back task

For each participant, the minimum time required to accurately process two ongoing tasks—n‐back letter detection and parity number decision—was determined in a pretest during the first session, lasting approximately 35 min. During the task, digits (1−9) and letters (A, C, T, L, N, E, U, P) alternated on the screen. Participants pressed the space bar with their left hand when a letter matched the last seen, and used their right hand to indicate whether a digit was odd or even. The maximum processing speed capacity was defined as the fastest stimulus time duration (STD) that allowed at least 85% accuracy. The average fastest STD for our participants was 0.831 ± 0.160 SD. This measure of processing speed capacity was included in the factor analysis on cognitive task scores (see further in Factor Analysis).

To induce cognitive load and fatigue before language exposure in Sessions 2 or 3 (Fig. 1), participants performed the dual task at their fastest predetermined STD, which requires sustained attentional control and induces cognitive fatigue (Borragán et al., 2017). The task script, run in Matlab2018b/Psychtoolbox via the Gorilla experiment builder, is available on osf.io/ay6er (Borragán et al., 2017). Participants performed this task for 16 min. Severity of fatigue was measured prior and post the cognitive fatigue manipulation (Fig. 1) by the VAS‐f (Lee, Hicks & Nino‐Murcia, 1991), which is a self‐rating scale and provides a quantifiable score of perceived subjective fatigue level. The scale consists of a fatigue subscale (13 items), used in the present study, and an energy subscale (5 items). On each item, participants were asked to rate from 0 to 10 what they felt at that moment (e.g., I feel exhausted, I feel sleepy, etc.). Sum scores across the items were calculated for each participant (see Table S2 for individual sum scores). The Cronbach's alpha for the items used in the current study was between 0.94 (Session 1) and 0.97 (Session 2) for both time points (prior and post cognitive fatigue manipulation), suggesting internal reliability consistency. A supplementary analysis (see “sanity check” and Fig. S1) confirmed that participants experienced greater fatigue after the TloadDack task compared to the control task (see Fig. S1).

#### 2.2.2 Additional cognitive tasks

##### 2.2.2.1 Digit span task

The digit span task was used to assess participants’ verbal working memory capacity. In this task, participants repeat increasingly longer sequences of digits in either forward (right) or backward (reverse) order. Forward span measures storage and maintenance capacity, while backward span also assesses the ability to manipulate and update information in short‐term memory (Ahmed et al., 2022). Each span consisted of 18 trials, where participants were presented with a sequence of digits (1−9) and recalled the sequence using a visually displayed numeric keyboard. Sequences started at two digits and increased to 10 digits, with two attempts per level. The digit span score was the longest sequence correctly recalled in at least one attempt. The task showed moderate internal consistency on item accuracy (Forward, KR‐20 = 0.49, split‐half *r* = .57, 95% CI [0.38, 0.74]; Backward: KR‐20 = 0.67, split‐half *r* = .71, 95% CI [0.54, 0.84]).

##### 2.2.2.2 Simon task

The Simon task measures stimulus‐response compatibility and is commonly used to study EFs, including inhibitory control and visuospatial attention (Cespón et al., 2020). In this task, participants respond to a nonspatial feature (e.g., color, shape) of a lateralized stimulus by pressing one of two buttons, each corresponding to a stimulus position. The task includes compatible (e.g., the word “left” on the left side of the screen) and incompatible (e.g., the word “left” on the right side) conditions. Reaction times (RTs) are longer, and accuracy is lower in the incompatible condition, a phenomenon known as the Simon Effect. In our experiment, participants completed 192 trials, with half of the trials being incompatible. They responded by pressing “F” for the word “left” and “J” for the word “right,” using their left and right index fingers, respectively. Trials had a 1500 ms response time limit, and feedback was given with thumbs‐up (correct) or thumbs‐down (incorrect/delayed) images. The Simon Effect was quantified as the difference in errors and RTs between compatible and incompatible trials. The task showed good internal consistency on item accuracy (KR‐20 = 0.75, *r* = .85, 95% CI [0.77, 0.91]).

##### 2.2.2.3 Switch task

Task switching, a key aspect of cognitive flexibility (Vandierendonck et al., 2010), involves shifting attention between tasks. In typical paradigms, participants alternate between two simple tasks, using a rule or cue to determine which task to perform. Switch trials, following a task change, are usually slower and/or less accurate than repeat trials, reflecting a “switch cost.” In our experiment, participants completed 97 trials, where they responded to either the color or shape of a figure based on its location. If the shape was at the top of the screen, they responded to the color (pressing “F” for blue and “J” for green). If at the bottom, they responded to the shape (pressing “F” for square and “J” for rectangular). The tasks alternated in a “repeat‐switch” pattern, with a 1500 ms response time limit. Feedback was given with thumbs‐up (correct) or thumbs‐down (incorrect) images for 200 ms. The difference in response times (for correct responses) and errors between switch and repeat trials was used to measure the switch cost. The task showed good internal consistency on item accuracy (KR‐20 = 0.78, *r* = .82, 95% CI [0.72, 0.89]).

#### 2.2.3 Exposure

Participants were exposed to 12 novel words across four structured syllable streams (languages) over two sessions. Each session included two streams with three repeating syllable triplets (i.e., “words”), constructed from 36 unique syllables with a consonant‐vowel structure, matched for bigram frequency in Dutch. No triplets had existing Dutch neighbors, as verified with WordGen (Duyck et al., 2004). A full list of syllables and their frequencies is provided in Table S3. Syllables were recorded by a native Dutch speaker and edited to a consistent 222 ms duration.

The triplets were repeated 90 times in a pseudorandom order in ∼1‐min clips, with TPs set at 100% within triplets and 33% between triplets (allowing one repetition of the same word, similar to Boeve et al., 2024, e.g., in language L1, /fi/ was always followed by /mo/, which was always followed by /ti/, while /ti/ could be equally followed by /fi/, /da/, or /sa/). Each stream was presented three times per session, resulting in 270 repetitions of each word. The exposure clips were alternated (Fig. 1), and all words were tested in a word recognition task after a short break.

The articulatory suppression task (continuous whispering) during the clips of one stream was practiced beforehand, where participants recorded themselves whispering “tah” at 4.5 Hz after listening to an example. During exposure, participants were prompted on the screen to either “whisper tah” or “listen only” while attending to the stream clips (see Fig. 1).

#### 2.2.4 Word recognition task

On each trial, participants first viewed a fixation cross for 500 ms, followed by two audio files with a 1500 ms interstimulus interval. One file contained a triplet from the exposure language, and the other a foil triplet. The foils were partwords crossing word boundaries in the language streams (e.g., for L1: ti‐da‐mu, ri‐sa‐ro). Each triplet was paired exhaustively with each foil using a Latin square design, with counterbalanced positions for the triplet (first or second presentation). This resulted in 18 recognition trials per language and 36 trials per session. Trials were randomly presented to each participant. Participants indicated first which structure sounded most familiar and then rated their memory (1: “I guessed,” 2: “It sounds familiar to the exposure,” 3: “I remember the structure from the exposure”). There was no time limit, and the next trial started after a response. The task showed moderate internal consistency for each test condition, similar to what is typically seen in adults (Arnon, 2020; Control condition: split‐half *r* = .52, 95% CI [0.33, 0.69], Whisper condition: split‐half *r* = .57, 95% CI [0.36, 0.74]; Fatigue condition: split‐half *r* = .58, 95% CI [0.43, 0.72]).

### 2.3 Statistical analyses

#### 2.3.1 Factor analysis and clustering approach

To extract a common measure representing performance on the higher cognitive (executive functioning) tasks, we used Maximum Likelihood Exploratory Factor Analysis (ML EFA). We opted for EFA to identify latent constructs that capture the specific set of cognitive measures we have available while remaining agnostic about the exact structure of these components. The ML approach was selected because it allows for the computation of various goodness‐of‐fit indices, such as the Root Mean Square Error of Approximation, which are unavailable in principal component analysis approaches. This method is also recommended by Pedraza et al. (2024).

First, we examined the potential structure of our set of individual cognitive measures in a data‐driven manner to extract a reliable common factor that could help distinguish between high and low CR groups. The cognitive measures included processing speed capacity on the TloadD Task, the forward span, the backward span, and the Simon and Switch cost (both in response time and errors).

To aid interpretation, individual scores were first transformed into z‐scores and then rescaled to a normal score with a mean of 100 and a standard deviation of 15, so that higher values represented better cognitive performance across all measures. The individual scores for the cognitive tasks used in the factor analysis are presented in Table S4. A backward digit span score is missing in two participants, and a Simon response time is missing in one participant, which were replaced by the mean norm score for the analyses.

To investigate the factor structure, we performed an exploratory maximum likelihood factor analysis with Varimax rotation (fa() function) using the psych package (Revelle, 2023) in R software (R Core Team, 2024). To assess the factorability of the data, we first inspected the off‐diagonal elements of the anti‐image correlation matrix. Variables with off‐diagonal correlations below 0.50 were deleted one by one using the antiImage() function. Next, we computed the Kaiser−Meyer−Olkin (KMO) test of sampling adequacy using a custom function, aiming for a KMO index of at least 0.60 to determine whether the data were suitable for factor analysis. We also performed Bartlett's test of sphericity (cortest.bartlett()) to test the hypothesis that the sample correlation matrix originated from a multivariate normal population where the variables are independent. Rejection of the null hypothesis indicates that the data are appropriate for extracting a common factor.

To ensure that a single factor could be reliably extracted, we conducted Horn's parallel analysis using the fa.parallel() function, which compares the eigenvalues of the observed data with those of randomly generated data at the 95th percentile. The parallel analysis suggested that one factor should be retained, which was supported by visual inspection of the eigenvalues in the plot produced.

We extracted the common factor using Maximum Likelihood extraction (fa() with fm = “ml”), followed by Varimax rotation to facilitate interpretation of the factor loadings. The extracted factor scores were saved as a new variable (efml) in the dataset (EFdata) for each participant. These participant‐level factor scores were then used in subsequent cluster analysis to categorize participants into high and low CR groups.

To define the high and low CR groups, we applied a K‐means clustering algorithm using a squared Euclidean distance metric, specifying two clusters based on the factor scores obtained from the exploratory factor analysis (EFA). The algorithm was run with a set seed to ensure reproducibility and used a maximum of 10 iterations. For each cluster (low and high CRs), we fitted a normal distribution with the corresponding mean (*µ*) and standard deviation (*σ*) for each cluster. The thresholds for defining the low and high groups were set as *T_low_ = µ_lower_+ σ_lower_* for the lower bound of the low group, and *T_high_ = µ_higher_* *− σ_higher_* for the upper bound of the high group. Participants whose factor score fell below *T_low_* or above *T_high_* were retained for the main group analyses. This classification approach ensured that participants were divided into two distinct groups based on their executive functioning level.

#### 2.3.2 Main analyses on statistical language learning

We used an alpha level of 0.05 for all statistical tests. Participant characteristics were compared across groups using two‐tailed independent *t*‐tests with Cohen's *d* effect sizes, with Hedges’ correction. Welch *t*‐tests were used when the two samples exhibited unequal variances. The word recognition data were analyzed using (generalized) linear mixed effects modeling with the lme4 package (Bates, Mächler, Bolker, & Walker, 2015) and the afex package (Singmann, Bolker, Westfall, Aust, & Ben‐Shachar, 2015) in R (R Core Team, 2024). We always aimed to include the maximal random effects structure for both participant and item factors, justified by the experimental design. However, in cases of convergence issues (e.g., singular fits), the maximal model was refitted by first removing the correlations among random slopes. Next, the highest‐order random slopes with the least estimated variance were removed (Singmann & Kellen, 2019). For investigating the effects of Cognitive Fatigue and Articulatory Suppression, the outcome variable was accuracy (binomial: 1 = correct, 0 = incorrect). The factors in the model included CR group (effect‐coded, with low CR group set to −1), Cognitive Fatigue or Articulatory Suppression (effect‐coded, with control set to −1), and their interaction. The final converging models were Accuracy ∼ Group × Cognitive Fatigue + (1+ Fatigue∣Subject) + (1+ Fatigue∣Word) for studying the effect of Fatigue, and Accuracy ∼ Group × Articulatory Suppression + (1+ Articulatory Suppression∣Subject) + (1+ Articulatory Suppression∣Word) for studying the effect of Articulatory Suppression.

We additionally explored group differences in implicit versus explicit knowledge acquisition as a function of cognitive fatigue and/or articulatory suppression. First, we tested whether participants performed above chance on guess trials (indicating implicit memory) across the different conditions using one‐sample *t*‐tests, which were further confirmed by using linear mixed effects models on accuracy scores (Group, Memory judgment, Cognitive fatigue/Articulatory suppression as predictor variables). Next, we tested whether participants built memory strength (“explicit knowledge”) for the words by regressing participants’ item‐specific memory judgment scores (1 for “guessed” to 3 for “remembered”) on accuracy (correct vs. incorrect). Memory strength is indicated by an increase in memory judgment from incorrect to correct trials (i.e., confidently “remembering” correct words), which was compared across groups and conditions. The final converging models for these additional analyses are presented as a footnote in the Results section.

The *p*‐values for the main effects and interactions were derived using the Wald Chi‐square test in the *Anova* function of the *car* package (Type III *p*‐values) (Fox & Weisberg, 2023). Pairwise comparisons were performed using least squares means using the *lsmeans* function from the *lsmeans* package (Lenth, 2016). We examined interactions both within and across levels of our factors, followed by post‐hoc tests with pairwise comparisons for each combination of factors using the *emmeans* package (Lenth, 2022). Additionally, effect sizes for each comparison were computed using the *eff_size* function in the *emmeans* package. Specifically, the standardized mean differences (Cohen's *d*) for each contrast were calculated, adjusting for the residual standard deviation (sigma) and residual degrees of freedom (edf) derived from the fitted model.

#### 2.3.3 Power analysis

Our primary hypothesis concerns a Group × Condition interaction. An a priori power analysis (G*Power 3.1.9.7; ANOVA: repeated measures, within–between interaction) with α = 0.05 and target power = 0.80, anchoring on a small‐to‐medium effect size (partial eta squared = 0.06 or *f* = 0.25), Number of groups = 2, Number of measurements = 2 with *r* = .50 and *ε* = 1.00, indicated *N* = 34 required. A sensitivity analysis shows that with our final *N* = 49 (min. subgroup *n* = 19, see below), the minimum interaction effect detectable with 80% power is *f* = 0.20; equivalently, with *N* = 49, we have 71% power for *f* = 0.18, 77% power for *f* = 0.20, 84% for *f* = 0.22, and >99% for an optimistic *f* = 0.31.

To align with our conducted mixed‐effects confirmatory analysis, we also ran a post‐hoc simulation‐based power analysis (1000 simulations) mirroring the planned model, which yielded comparable power thresholds, with 78% power for our observed effects. The script for that analysis is available in our data respository.

#### 2.3.4 Reliability analyses

Internal reliability and item consistency in all relevant tasks were evaluated using Cronbach's α (or the equivalent KR‐20 for binary items) and split‐half reliability. These were computed with the alpha() and splitHalf() functions from the *psych* package in R (Revelle, 2025). The split‐half function partitions items into two halves across multiple random splits, computes the correlation between the halves, and applies the Spearman–Brown correction. For each task, KR‐20 and average split‐half reliability estimates (with 95% confidence intervals) are reported. Because items in the statistical learning task were counterbalanced across participants and conditions, classical internal‐consistency measures such as Cronbach's α/KR‐20—which assume an identical set of items across all participants—are not appropriate. Therefore, only split‐half coefficients are reported for this task. We calculated them separately for each learning condition: control, under cognitive fatigue, and under articulatory suppression. Please note that our primary analyses are group‐based comparisons on statistical learning. Hence, while reporting internal consistency is informative, achieving high reliability is not strictly necessary to support our conclusions, as the statistical tests compare aggregate group performance rather than individual scores.

## 3 Results

### 3.1 Factor analysis on cognitive measures

Individual distributions of the cognitive measures (i.e., normal scores for processing speed, forward span, backward span, Simon and switch cost in errors, and response time) and their bivariate correlations are presented in Fig. S2.

We first aimed to identify a common factor across our measures, following the approach of Pedraza et al. (2024). The diagonals of the anti‐image correlation matrix were all above 0.5 after iteratively removing the processing speed measure derived from the TloadDback pretest, the Switch Cost measures (response time and error), and the Simon Cost error measure. The final matrix yielded a KMO measure of sampling adequacy of 0.60 and a significant Bartlett's test of sphericity (*χ* ^2^(3) = 9.4, *p* = .024), suggesting that the remaining measures (forward and backward span, and Simon Cost in response time) were suitable for factor analysis (see also the correlation matrix in Fig. S2). Parallel analysis indicated that from these data, one common factor could be extracted, as shown in Fig. S3. The distribution across participants of the extracted factor score is also presented there. A two‐means cluster analysis yielded two distinct groups: 19 participants in a low CR group and 24 participants in a high CR group (see the bimodal distribution in Fig. S3). Their cognitive performance is summarized in Table S4. Because the group classification was based on an extracted latent factor capturing shared variance on the digit span tasks and the Simon task, the resulting groups are expected to differ primarily on these measures rather than across all cognitive tasks.

### 3.2 Statistical learning performance

Statistical learning performance, defined as above‐chance accuracy on the word recognition task, as a function of cognitive fatigue and articulatory suppression, is presented in Table 1. Participants in both the high and low CR groups performed above chance in all conditions.

**Table 1:** Overall statistical learning performance

	*Mean*	*SD*	*SE*	*t*	*p*	*Effect size*
**Low EF (*N* = 19)**						
No Cognitive Fatigue						
No articulatory suppression	76.3	13.8	3.2	8.3	***	1.8
Articulatory suppression	62.3	16.1	3.7	3.3	**	0.73
Cognitive Fatigue						
No articulatory suppression	71.9	15.9	3.6	6.0	***	1.3
Articulatory suppression	63.5	14.4	3.3	4.1	***	0.89
**High EF (*N* = 24)**						
No Cognitive Fatigue						
No articulatory suppression	70.4	13.8	2.8	7.2	***	1.4
Articulatory suppression	64.8	14.9	3.0	4.9	***	0.96
Cognitive Fatigue						
No articulatory suppression	75.2	13.0	2.7	9.5	***	1.9
Articulatory suppression	66.9	18.0	3.7	4.6	***	0.91

*p < 0.05

**p < 0.01

***p < 0.001

#### 3.2.1 Individuals with high CRs are less efficient statistical learners and benefit more from cognitive fatigue than people with low CRs

**Fig. 2:**
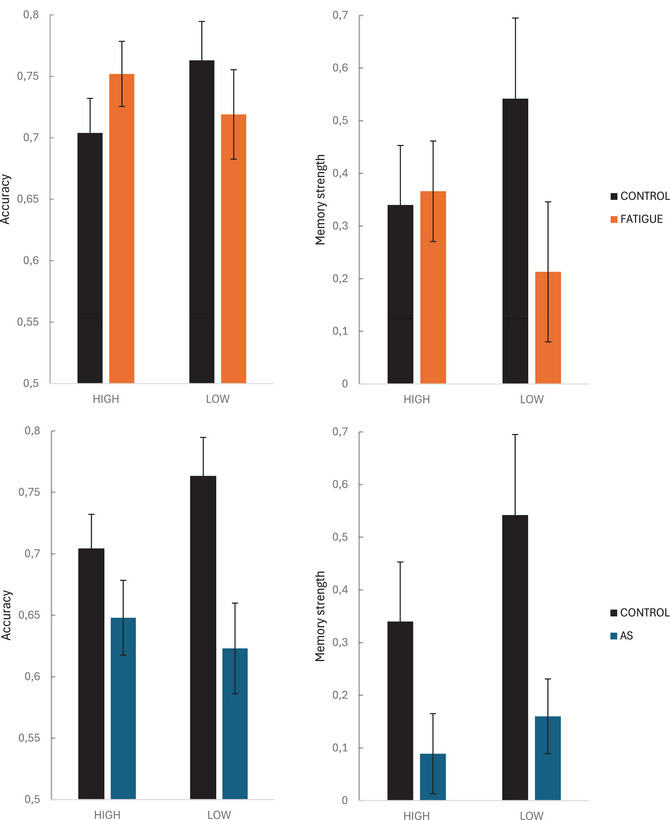
Statistical learning performance (word recognition accuracy) and explicit knowledge (memory strength) as a function of cognitive fatigue (upper panel) and articulatory suppression (AS) (lower panel) in high versus low CR groups.

When treating the cognitive factor score first as a continuous variable, a significant interaction between the extracted factor score and cognitive fatigue appeared (i.e., factor score × Cognitive Fatigue: *b* = −0.18, *SE* = 0.09, *z* = −2.12, *p* = .034).^2^ Importantly, this analysis includes the full range of cognitive factor scores across all 49 participants. Our main mixed effects analysis, which used CR group as a categorical factor based on extreme groups (high vs. low CRs) identified through a cluster analysis, showed that cognitive fatigue differently affected accuracy on the word recognition task in low versus high CR group (i.e., Group × Cognitive Fatigue: *b* = −0.19, *SE* = 0.07, *z* = −2.83; *X* ^2^(1) = 8.01, *p*  = .005). This interaction is visualized in Fig. 2A. Post‐hoc pairwise comparisons for the interaction showed that while the high CR group showed lower accuracy than the low CR group in the control condition (*p* = .022, *d* = −0.5), this group difference disappeared under cognitive fatigue (*z*<1). Cognitive fatigue improved performance in the high CR group (*p*_one‐tailed_ = .023, *d* = 0.4) but not in the low CR group (*p* = .096, *d* = −0.4).

#### 3.2.2 Cognitive fatigue decreases explicit memory strength in people with low CRs

Statistical learning performance as a function of memory judgment is presented in Table 2. According to a guessing criterion (Dienes & Berry, 1997), above‐chance performance on guess trials would indicate implicit memory and is difficult to observe in adults’ statistical learning outcomes when these are assessed using the word recognition task (see also Batterink et al., 2015; Smalle et al., 2022). In the current study, the low CR group showed above‐chance performance on guess trials in the fatigue condition, indicating implicit memory formation when fatigued during the exposure (Memory Judgment × Fatigue in the low group: *b* = 0.46, *SE* = 0.22, *z* = 2.1, *p* = .038; Memory Judgment × Fatigue × Group*: b* = −0.39, *SE* = 0.14, *z* = −2.7; *X* ^2^_(2)_ = 7.8, *p* = .019).^3^

**Table 2:** Statistical learning performance as a function of memory judgment

	*Mean*	*SD*	*SE*	*t* ^a^	*p*	*Effect size*
**Low EF (*N* = 19)**						
Control						
Guess (11% trials)	55.2	37.6	10.1	<1		
Familiar (49% trials)	72.2	19.9	4.6	4.9	^***^	1.06
Remember (40% trials)	84.0	27.9	6.6	5.2	^***^	1.16

Cognitive Fatigue						
Guess (13% trials)	68.2	37.9	9.2	2.0	^*^	0.46
Familiar (57% trials)	69.7	14.9	3.4	5.8	^***^	1.3
Remember (30% trials)	80.9	29.7	7.2	4.3	^***^	0.99

Articulatory Suppression						
Guess (29% trials)	53.7	24.9	5.7	<1		
Familiar (59% trials)	64.0	21.3	4.9	2.9	^**^	0.63
Remember (12% trials)	73.0	28.6	7.7	3.0	^**^	0.75

**High EF (*N* = 24)**						
Control						
Guess (19% trials)	53.0	40.7	8.4	<1		
Familiar (45% trials)	68.9	24.4	5.0	3.8	^***^	0.75
Remember (36% trials)	76.3	31.0	6.6	4.0	^***^	0.82

Cognitive Fatigue						
Guess (11% trials)	50.4	39.4	9.9	<1		
Familiar (46% trials)	69.4	20.3	4.2	4.7	^***^	0.92
Remember (43% trials)	81.4	20.2	4.3	7.2	^***^	1.46

Articulatory Suppression						
Guess (24% trials)	54.4	36.6	7.5	<1		
Familiar (58% trials)	66.6	19.1	3.9	4.3	^***^	0.84
Remember (18% trials)	74.5	25.2	5.8	4.2	^***^	0.93

^a^ One‐sample *t*‐tests are reported.

*p < 0.05

**p < 0.01

***p < 0.001

Mixed effects analysis on memory strength showed explicit word knowledge in both the low (correct vs. incorrect, *p* = .005, *d* = 0.3) and high group (*p* = .048, *d* = 0.2) in the control condition (Group × Correct: *b* = 0.04, *SE* = 0.043, *t*<1). While explicit knowledge disappeared in the fatigue condition for the low group (Correct vs. Incorrect: *p* = .52, *d* < 0.1; Fatigue × Correct: *b* = −0.07, *SE* = 0.027, *t* = −2.6, *p* = .01 (Fig. 2B), the Fatigue × Correct effect in the high group was not significant (*b* = 0.01, *SE* = 0.023, *t* < 1), with a significant Group × Fatigue × Correct interaction indicating that fatigue modulated memory strength differently across groups (*b* = 0.04, *SE* = 0.018, *t* = 2.3; χ^2^_(1)_ = 5.2, *p* = .023).^4^

#### 3.2.3 Individuals with low CRs are affected more by articulatory suppression than people with high CRs

There was no significant interaction between the extracted factor score and articulatory suppression (i.e., factor score × Articulatory Suppression: *b* = −0.084, *SE* = 0.09, *z*<1). However, there was a main effect of Articulatory Suppression (i.e., *b* = 0.25, *SE* = 0.13, *z* = 1.9, *p* = .05).^5^ Mixed effects analysis with CR group as factor showed that articulatory suppression differently affected accuracy in the word recognition task in low versus high CR group (i.e., Group × Articulatory Suppression: *b* = −0.17, *SE* = 0.067, *z* = −2.52; *X* ^2^(1) = 6.34, *p* = .011).^6^ The interaction is visualized in Fig. 2C. The group difference seen in the control condition (see earlier) disappeared under articulatory suppression (*p* = .49, *d* = 0.1). Articulatory suppression impaired performance in the low CR group (*p*_one‐tailed_ = .003, *d* = 0.9) but not significantly in the high CR group (*p*_one‐tailed_ = .2, *d* = 0.24).

#### 3.2.4 Articulatory suppression decreases explicit memory strength in both groups

Articulatory suppression decreased explicit knowledge (Correct × Motor: *b* = −0.04, *SE* = 0.017, *t* =  −2.7; *X* ^2^_(1)_ = 7.2, *p* = .007) equally in both groups (Group × Motor × Correct: *b* = 0.01, *SE* = 0.017, *t<*1). No explicit knowledge was acquired from exposure streams during whispering in either group (correct vs. incorrect: low: *p* = .3, *d*<0.1, high: *t*<0.1).

#### 3.2.5 Spontaneous speech synchronization during language exposure

**Fig. 3:**
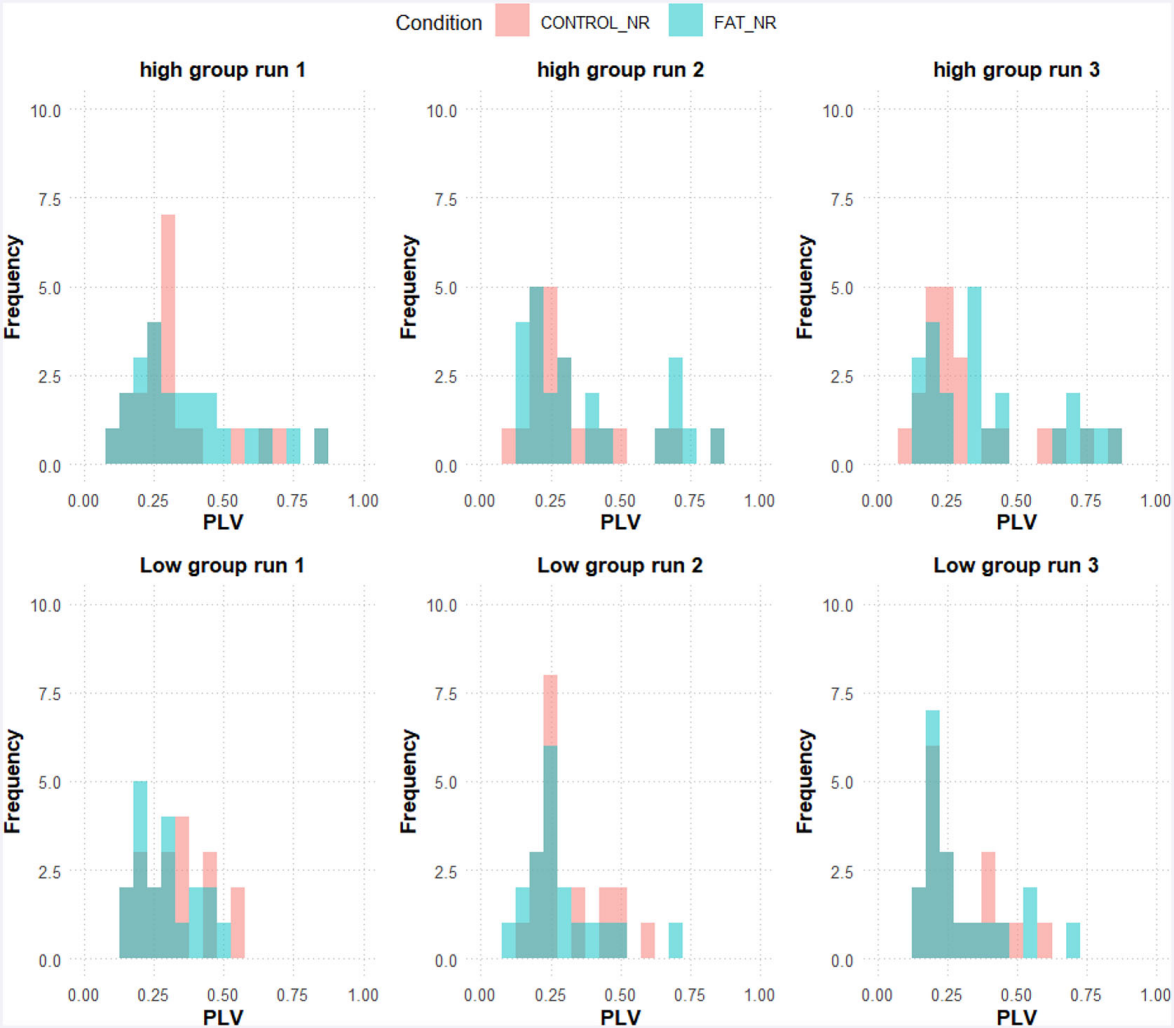
Distribution of the phase‐locking values across three whispering runs, separately for each CR group (high: upper panel; low: lower panel) and as a function of cognitive fatigue (pink: no cognitive fatigue induction; blue: cognitive fatigue induction).

The phase locking values for speech synchronization during whispering are presented in Table 3, and its distribution across participants is visualized in Fig. 3. The high CR group synchronized slightly better under fatigue (*p*_one‐tailed_ = .041, *d* <1) than the low CR group (*t* <1); however, no significant group effects appeared (Group: *X* ^2^_(1)_<1, Fatigue: *X* ^2^_(1)_<1; Group × Fatigue: *b* = −0.01, *SE* = 0.0085, *t* =  −1.62; *X* ^2^_(1)_ = 2.62, *p* = .11).^7^

**Table 3:** Average phase‐locking values for participants’ spontaneous speech synchronization during the Articulatory Suppression task across the three language clips/runs (see Fig. 1; condition)

	Low EF (*N* = 19)			High EF (*N* = 24)		
	*Mean*	*SD*	*SE*	*Mean*	*SD*	*SE*
Run 1						
Control	0.320	0.12	0.028	0.336	0.19	0.040
Cognitive Fatigue	0.292	0.10	0.024	0.362	0.20	0.041

Run 2						
Control	0.314	0.13	0.031	0.334	0.19	0.041
Cognitive Fatigue	0.287	0.14	0.032	0.375	0.23	0.048

Run 3						
Control	0.298	0.13	0.031	0.334	0.20	0.043
Cognitive Fatigue	0.304	0.16	0.036	0.403	0.23	0.049

Averaged						
Control	0.311	0.13	0.017	0.335	0.19	0.023
Cognitive Fatigue	0.294	0.13	0.018	0.380	0.22	0.026

## 4 Discussion

Previous recent research has shown that cognitive depletion in adults enhances statistical language learning (e.g., Smalle et al., 2022), suggesting that adults’ top‐down cognitive control constrains effortless learning processes typical of infant language acquisition (cognitive cost hypothesis; Smalle & Möttönen, 2023). Separately from this research, accumulating evidence suggests that the left speech motor system—especially its interaction (synchronization) with auditory processing—supports adults’ statistical language learning ability (Assaneo et al., 2019; Boeve et al., 2024; Orpella et al., 2022). The present study aimed to integrate these two research lines by examining within adults (1) how individual differences in CRs influence statistical language learning and (2) how high‐level, domain‐general cognitive and low‐level, language‐specific articulatory mechanisms interact during this learning.

Consistent with a cognitive cost hypothesis, our results show that individuals with lower CRs—measured via a latent factor underlying forward/backward span and the Simon task performance (similar to Pedraza et al., 2024)—outperformed those with higher CRs in a post‐exposure word recognition task, which is typically used to assess statistical language learning (Batterink, Paller, & Reber, 2019). These individuals also formed stronger memory representations of the learned words, as indicated by higher confidence in remembering correct words. This pattern suggests that weak top‐down control is associated with efficient statistical language learning ability, in line with dual‐process models for statistical learning (e.g., Daw et al., 2005; Pedraza et al., 2024).

Interestingly, in individuals with higher CRs, statistical language learning ability was enhanced under cognitive fatigue, although their memory strength remained unchanged. This finding extends the cognitive cost hypothesis, previously only assessed in between‐subject designs, indicating that temporarily reducing cognitive control via fatigue can enhance within‐subject learning efficiency—especially in those with high CRs. Recent work in the perceptual‐motor domain suggests that within‐subject states of mind‐wandering and/or local sleep in which specific brain regions transiently go “offline” can be beneficial for implicit, statistical learning. For instance, mind wandering has been shown to facilitate the extraction of hidden regularities (Vékony et al., 2025), and this benefit has been linked to increases in periodic electroencephalography (EEG) activity (e.g., alpha/theta rhythms) which reflect disengagement from external task demands and a shift toward internally oriented, pattern‐extraction–conducive brain states (Simor et al., 2025). From this perspective, cognitive fatigue in the present study may have promoted mind wandering—a neurocognitive state that is particularly suited for statistical learning. It can be speculated that the high‐CR individuals spontaneously mind‐wander less than low‐CR individuals, and that cognitive fatigue especially enhances spontaneous mind wandering in high‐CR individuals, explaining the facilitatory effect of cognitive fatigue on statistical learning in our high CR, but not low CR, group. However, further studies are needed to test whether cognitive fatigue enhances mind wandering and whether mind wandering is beneficial for statistical language learning.

It is important to note that, unexpectedly, cognitive fatigue did not increase the efficiency of statistical language learning and memory strength in the low CR group. Based on the cognitive cost hypothesis, we would predict an overall positive effect of cognitive fatigue on adult statistical learning, albeit perhaps smaller in people with weaker CRs. The absence of a positive effect and trend toward a negative effect may suggest that a minimal level of attentional and cognitive control remains necessary for statistical learning to occur (at least with respect to memory storage; see for instance, Batterink & Paller, 2019). This interpretation aligns with cross‐domain evidence that attention modulates statistical learning, albeit with some differences. For example, in visual statistical learning, at least some attentional resources—such as sustained attention—are necessary for extracting regularities during exposure (Duncan, van Moorselaar, & Theeuwes, 2025), whereas offline memory storage appears less dependent on attentional fluctuations during exposure (Zhang & Rosenberg, 2024). Visual statistical learning can also occur in the absence of explicit top‐down (i.e., goal‐oriented) attention (Duncan & Theeuwes, 2020). In contrast, in statistical language learning, particularly speech segmentation, focused attention is critical for memory storage: performance on word recognition tasks drops to chance levels without attention during speech exposure (Toro, Sinnett, & Soto‐Faraco, 2005; Papoutsi, Zimianiti, & Bosker, 2024), but memory can still be formed without top‐down attentional control such as conscious awareness of the learning material (Arciuli, Torkildsen, Stevens, & Simpson, 2014) or in the presence of a competing speech stream (i.e., cocktail party environment; Papoutsi et al., 2024). These findings suggest that in our low CR group, cognitive fatigue may have pushed their attentional resources below a critical threshold, tending to impair memory storage and overall learning efficiency. This view of basic CRs being engaged in statistical language learning also fits well with empirical work by Palmer and Mattys (2016). Using dual‐task paradigms, they showed that taxing working memory during exposure to continuous speech reduced the beneficial effect of a slower stimulus presentation rate on subsequent word recognition performance, an effect observed regardless of the linguistic nature of the secondary task. This indicates that sufficient (domain‐general) attentional and working‐memory resources support the maintenance and integration of distributional information, enabling learners to capitalize on input conditions that are favorable for memory encoding. Alternatively, the null effect and negative trend could reflect regression to the mean in this group, given their initially strong word recognition. However, it is important to note that our carefully counterbalanced design (see Fig. 1) likely mitigated potential regression to the mean, at least in part (Barnett, van der Pols, & Dobson, 2004). Nevertheless, the within‐subject improvement that we observe in the high‐resource individuals under fatigue provides compelling evidence that transient cognitive depletion can facilitate individual learning—aligning with the cognitive cost account.

The unchanged memory strength in the high CR group suggests that although depleting their cognitive system improved overall statistical learning efficiency (i.e., performance in the word recognition task), it did not affect their explicit memory for newly acquired linguistic knowledge (Batterink et al., 2015). Hence, the improvement in their overall learning outcomes as a result of cognitive fatigue is likely to reflect an improvement in learning that is not explicit, but perhaps implicit in nature. This latter could have been captured by additional processing‐based measures for statistical learning, such as directly via neural entrainment during exposure (e.g., Smalle et al., 2022; Batterink et al., 2019) or indirectly via serial‐induced recall (i.e., indirect; e.g., Isbilen et al., 2020; Kidd et al., 2020) and/or target detection (e.g., Batterink et al., 2015) after the exposure (during memory formation). Most studies investigating age‐related changes and/or other individual variabilities in statistical language learning do not find clear patterns of variance when looking at direct measures (e.g., Smalle & Bogaerts, 2024), but some differences appear when looking at indirect memory measures (for a review, see Forest, Schlichting, Duncan, & Finn, 2023). For instance, research in adults has shown that inducing cognitive fatigue only boosted the outcomes of statistical learning, that is, acquired word knowledge, but not the online learning itself (i.e., the trajectory of learning as measured via neural entrainment; Smalle et al., 2022). Similarly, child‐adult differences are not observed in online learning measures, but subtle differences emerge in some offline measures, particularly in the nature of the acquired knowledge (implicit vs. explicit; e.g., Moreau et al., 2022; Smalle & Bogaerts, 2024). This suggests that individual variance may become more apparent at later stages of learning, such as during memory formation, and more specifically in the nature of the learning outcome, rather than in the learning trajectory or extraction process itself.

Based on prior work, we used a guessing criterion to infer implicit knowledge in our participants’ word recognition performance (Batterink et al., 2015). Overall, we observed, in contradiction to an implicit learning account, no above‐chance performance on guess trials in the high‐resource group under fatigue. Instead, we found implicit knowledge only in the low‐resource group, whose overall learning efficiency and memory strength declined as a result of cognitive fatigue. A possible interpretation is that the overall word recognition accuracy score reflects a composite of implicit and explicit contributions (see, for instance, Batterink et al., 2019), and that the observed decline in memory strength under fatigue in the low group reflects a selective strong drop in explicit word knowledge, while the above‐chance performance on guess trials reflects a selective though weak unlocking of some implicit knowledge (not strong enough to improve overall word recognition outcomes). The contrasting finding that in the high‐resource group, cognitive fatigue led to better word recognition without any measurable change in memory strength or implicit knowledge assessed by the guessing criterion opens two possible explanations: (1) we may have failed to detect implicit learning within our memory measure‐at‐hand due to methodological limitations—for example, too few trials to reliably capture guessing accuracy, as some work suggests that a minimum of four trials is needed (Pollet & Little, 2017), and in the current study, we only observed a participant average of 3.9 ± 2.8_SD_ guess trials per condition (control condition: 3.1 ± 2.3 _SD_, AS condition: 4.9 ± 3.02 _SD_, fatigue condition: 2.6 ± 1.6 _SD_); and/or (2) their improved performance as a function of fatigue stems from another underlying mechanism not captured by our current measures—such as increased reliance on perceptual salience, encoding, and/or speech‐motor synchronization during the exposure. Regarding the latter, when calculating phase‐locking values for potential spontaneous speech synchronization during the whispering tasks (articulatory suppression) in the experiment, we observe increased synchrony under fatigue in the high group, in line with such view. However, we need to be cautious in overinterpreting this finding as no significant interaction effect on phase‐locking values was revealed.

Articulatory suppression during speech exposure disrupted our participants’ learning outcomes—both in terms of word recognition and memory strength. Interestingly, it affected the people with lower CRs more strongly. Orpella et al. (2022) showed that a left frontoparietal network is affected by articulatory suppression during speech exposure, and that this is the network that supports statistical language learning in people with high spontaneous speech synchrony abilities. As postulated in their discussion, activity in the left frontoparietal network during statistical learning could be associated with a temporal attention mechanism, which might have been disrupted more strongly by cognitive fatigue in our participants with low CRs. This would be in accordance with our idea of a minimal level of attentional control that is needed for statistical learning (see above), and which might have been affected by cognitive fatigue and articulatory suppression in the low CR group specifically. Alternatively, the articulatory suppression effect in the low CR group may suggest a compensatory reliance on articulatory mechanisms when top‐down support is limited; although such compensation was not reflected in these participants’ spontaneous speech‐motor synchrony (higher phase locking values in the low group than the high group).

Overall, together, our findings provide new evidence on how distinct memory systems and learning mechanisms interact to support statistical language learning. Crucially, our results highlight the value of integrating the dynamic role of both domain‐general *cognitive* and domain‐specific *motor mechanisms* underlying statistical language learning, which have typically been investigated in isolation. Based on our data, we tentatively propose that the adult brain imposes a processing *bottleneck*, whereby stronger top‐down cognitive control can inhibit access to implicit learning and its underlying speech‐motor mechanisms that support statistical learning, thereby shaping individual variability in learning efficiency. However, this proposal requires further empirical support through more comprehensive research. For instance, future work should more thoroughly investigate which higher‐cognitive abilities constrain learning and underlying speech‐motor mechanisms, and how this varies at different language development stages across individuals (also in atypical development).

Developing a framework that considers multiple interacting sources of variance in statistical learning may help reconcile inconsistencies in the literature linking statistical learning, CRs, and language outcomes (e.g., Bogaerts et al., 2022). Although there is general consensus on developmental trajectories of cognitive control and language acquisition, consensus on the developmental trajectory of statistical learning is lacking: some studies support age‐invariant models, while others suggest it improves early in life, declines in childhood, or continues to enhance through middle childhood (e.g., Zwart et al., 2019). This variability challenges the ability of the current statistical‐learning theory to explain maturational constraints in language development (Forest et al., 2023; Thiessen et al., 2016) and capture atypical language trajectories in children with developmental disorders such as autism or specific language impairments (Saffran, 2018; Zwart et al., 2019).

Importantly, our data do not imply that reduced CRs directly explain children's superior language learning, nor do they provide evidence for critical period mechanisms. Rather, they underscore the need to distinguish factors (e.g., EFs) that may constrain implicit, effortless learning from those that can support it (e.g., auditory–motor coupling). Identifying these factors, and how they vary across individuals and developmental profiles, will not only advance theoretical understanding of variance in language acquisition, but also opens opportunities for understanding atypical language development and tailoring interventions to individual needs. For instance, children with developmental language disorder often present deficits in both procedural/statistical learning and EFs (Ullman & Pierpont, 2005). Building on this, Baron and Arbel (2022) propose that interventions should be tailored along a personalized implicit–explicit continuum, depending on which learning systems can be most effectively engaged. Our data, showing that explicit, higher‐cognitive factors can constrain and implicit, lower‐level speech‐motor factors enhance effortless language learning, depending on the cognitive profile of the individual, could offer new opportunities for such targeted interventions.

As mentioned in the introduction, adult learners bring substantial prior linguistic knowledge (related to their first language) to a language learning task (cf. entrenchment hypothesis). While freeing up CRs may benefit initial stages of language learning—particularly for learning the raw probabilistic structure of speech (Elazar et al., 2022; Siegelman et al., 2018)—these model‐free processes can become rigid or maladaptive when the environment changes. This limitation is especially evident in adults acquiring a second language, where existing representations can constrain adaptation to novel patterns (Finn & Kam, 2008; LaCross, 2015). In such situations, successful learning requires a constant process of updating: modifying previously acquired probabilistic representations to align with the new structures. Recent work from Pedraza et al. (2025) in the perceptual‐motor domain shows a positive relationship between updating statistical regularities and inhibitory control, but a negative relationship with semantic fluency (i.e., access to long‐term memory representations). This suggests that, in contrast to acquiring raw probabilistic patterns, updating them might be a multifaceted cognitive process that requires some aspects of cognitive control, such as inhibition, as well as some degree of model‐free functioning (Pedraza et al., 2025). This raises an important question: to what extent do top‐down CRs constrain early stages in second‐language learning, when previously learned patterns must be substantially modified? Investigating both the cognitive cost and linguistic entrenchment hypotheses could be an important future avenue in developing our framework. It is essential to understand how EFs and statistical learning processes compete, cooperate, or operate independently in different conditions. Competition may arise when the novel language differs substantially from the learner's native language, such that prefrontal cortex–dependent control processes constrain the implicit extraction of unfamiliar speech regularities. In contrast, some cooperation may occur when the new language is more similar to the native language, allowing some executive resources, such as inhibitory control, to suppress prior knowledge and support the encoding, maintenance, and/or flexible updating of probabilistic representations without strong interference from entrenched first‐language patterns. This perspective highlights that differences in language learning are determined by a combination of cognitive and linguistic resources of the learner and the demands of the new learning environment.

Moreover, while our study focuses on adults, children differ from adults both in the degree of linguistic entrenchment and in the maturation of auditory‐motor and cognitive control systems. As a result, the relationship between CR‐related individual differences and statistical language learning is unlikely to be identical in children and adults. Future work should examine the CR−learning relationship in different age groups.

The present findings may have implications beyond speech and auditory–motor learning. Although our experiments targeted statistical language learning, the idea that CR allocation can modulate implicit learning could extend to other perceptual or sensorimotor domains of statistical learning. For example, sequential pattern learning in vision or motor control also requires the coordination of sensory input, prediction, and action (Pedraza, Vékony, & Nemeth, 2023) and can similarly be influenced by the availability of domain‐general resources (see Pedraza et al., 2024 for evidence). At the same time, the bottleneck we observe in high‐CR adults may be partly tied to the specific demands of auditory–motor speech processing, which differs from learning in other modalities and domains. We, therefore, view it as an important direction for future research to test whether analogous resource‐related constraints or motor restrictions arise in nonspeech implicit learning tasks. Investigating these cross‐modal parallels would help clarify the extent to which the mechanisms identified here reflect domain‐general principles versus modality‐specific processes.

Several limitations in our study warrant some consideration. First, our sample consisted exclusively of first‐year university students in Psychology—a relatively homogenous group in terms of age, education, and cognitive functioning, even in inclusive university settings (there are no entrance exams or grade criteria for psychology programs in Belgium). Future research should examine more diverse populations—both in terms of age and educational background—to better capture the range of cognitive and neural mechanisms involved in statistical learning. Second, the latent factor we used to index cognitive control was derived from a rather limited set of tasks (forward/backward span and the Simon task), which, while widely used, may not fully capture the complexity of executive functioning. It remains unclear which specific cognitive control processes (e.g., inhibition, updating, task switching) drive the observed effects. A more nuanced assessment of EFs—including broader task batteries and questionnaires—could help clarify the mechanisms underlying the cognitive cost hypothesis and provide deeper insight into which aspects of cognitive control are facilitative versus detrimental to statistical learning. We hope that our findings open important avenues for future research aiming to unpack how individual differences in the cognitive architecture shape the efficiency and nature of language learning in adults.

## Funding

The research was funded by a grant from the Research Foundation ‐ Flanders (Grant No. 1211421N, Project No. 3E091720) to the first author EHMS. EK and RM were supported by the Research Council of Finland (grant awarded to RM, 342979). RM was also supported by the Strategic Research Council (DALAI‐FIN, 373227).

## Conflict of interest

We state no conflict of interest.

## Supporting information



Supplementary Information

## Data Availability

Data and materials are available at https://osf.io/zp5ub/?view_only=20b3097e73ce41078d0b3725d43cd966.

## Notes

1 The spacing between sessions was determined by logistical constraints so that we could test as many participants as possible in an as short time as possible (i.e., 6 weeks) and at the same time leave enough time between the two learning sessions to avoid interference and awareness. Hence, we organized testing our participants according to a predetermined scheme (see Table S1). The scheduling structure resulted in approximately 2–3 days between Sessions 1 and 2 and 1 week between the two learning Sessions 2 and 3.

2 The converging model included random intercepts and slopes for the effect of *Fatigue* at the subject and word levels.

3 The converging model included a random intercept and slope for the effect of *Fatigue* at the subject level and a random intercept at the word level.

4 The converging model included random intercepts and slopes for the effect of *Fatigue* and *Accuracy* at the subject and word levels.

5 The converging model included a random intercept and slope for the effect of *Articulatory Suppression* and the *factor score* at the subject level and a random intercept and slope for the effect of *Articulatory Suppression* at the word level.

6 The converging model included a random intercept and slope for the effect of *Articulatory Suppression* at the subject and word level.

7 The converging model included random intercepts and slopes for the effect of *Fatigue* and run at the subject level.
