# Supplementary material for: The Effects of Cognitive Fatigue and Articulatory Suppression on Statistical Language Learning Depend on the Strength of Cognitive Resources
Source: Cogn Sci. 2026 Feb 23;50(2):e70178. doi: 10.1111/cogs.70178 (PMC12928678; doi:10.1111/cogs.70178)
Supplement: Supplementary file 1 — Supplementary Information [file COGS-50-e70178-s001.docx]

**Table S1. Participant Testing Scheme**

|  | Week 1 | Week 2 | Week 3 | Week 4 | Week 5 | Week 6 |
| --- | --- | --- | --- | --- | --- | --- |
| Monday | (am) Ps 1-4 (S1) | (pm) Ps 13-16 (S1) | (am) Ps 25-28 (S1) | (pm) Ps 37-40 (S1) | (am) Ps 49-50 (S1) |  |
| Tuesday | (am) Ps 5-8 (S1) | (am) Ps 17-20 (S1)  (pm) Ps 21-24 (S1) | (am) Ps 29-32 (S1)  (pm) Ps 33-36 (S1) | (am) Ps 41-44 (S1)  (pm) Ps 45-48 (S1) |  |  |
| Wednesday | (am) Ps 1-4 (S2)  (pm) Ps 9-12 (S1) | (am) Ps 1-4 (S3)  (pm) Ps 13-16 (S2) | (am) Ps 25-28 (S2)  (pm) Ps 13-16 (S3) | (am) Ps 25-28 (S3)  (pm) Ps 37-40 (S2) | (am) Ps 49-50 (S2)  (pm) Ps 37-40 (S3) | (am) Ps 49-50 (S3) |
| Thursday | (am) Ps 5-8 (S2) | Ps (am) 5-8 (S3)  (pm) Ps 21-24 (S2) | (am) Ps 29-32 (S2)  (pm) Ps 21-24 (S3) | (am) Ps 29-32 (S3)  (pm) Ps 45-48 (S2) | (pm) Ps 45-48 (S3) |  |
| Friday | (pm) Ps 9-12 (S2) | (am) Ps 17-20 (S2)  (pm) Ps 9-12 (S3) | (am) Ps 17-20 (S3)  (pm) Ps 33-36 (S2) | (am) Ps 41-44 (S2)  (pm) Ps 33-36 (S3) | (am) Ps 41-44 (S3) |  |

*Note.* Ps = Participants; S = Session. All participants were tested individually in the lab between 9am-5pm, reserving one hour per session per participant. For convenience, we present them here as morning (am) and afternoon (pm) participants.

**Table S2**: Individual sum scores on the Visual Analogue Scale for fatigue (VAS) questionnaire (VAS-f1 = score pre the cognitive fatigue task; VAS-f1 = score post the cognitive fatigue task; VAS-c1 and VAS-c1 = score pre and post the control task).

| **Participant** | **VAS-f1** | **VAS-f2** | **VAS-c1** | **VAS-c2** |
| --- | --- | --- | --- | --- |
| 2 | 11 | 13 | 9 |  |
| 3 | 33 | 42 | 30 |  |
| 4 | 20 | 71 | 43 | 44 |
| 5 | 47 | 53 | 79 | 76 |
| 6 | 14 | 44 | 8 | 13 |
| 7 | 31 | 31 | 54 | 58 |
| 8 | 19 | 28 | 15 | 14 |
| 9 | 31 | 34 | 18 | 19 |
| 10 | 31 | 46 | 10 | 13 |
| 11 | 56 | 49 | 55 | 60 |
| 12 | 44 | 39 | 52 | 46 |
| 13 | 39 | 38 | 22 | 12 |
| 14 | 40 | 40 | 13 | 22 |
| 15 | 12 | 18 | 12 | 13 |
| 16 | 9 | 6 | 14 | 4 |
| 17 | 9 | 10 | 25 | 20 |
| 18 | 21 | 28 | 42 | 39 |
| 19 | 28 | 27 | 8 | 7 |
| 20 | 44 | 46 | 44 | 33 |
| 21 | 113 | 116 | 103 | 104 |
| 22 | 28 | 26 | 20 | 14 |
| 23 | 29 | 30 | 53 | 57 |
| 24 | 40 | 37 | 61 | 47 |
| 25 | 48 | 43 | 52 | 48 |
| 26 | 18 | 10 | 3 | 1 |
| 27 | 30 | 55 | 20 | 12 |
| 28 | 24 | 47 | 10 | 5 |
| 29 | 28 | 40 | 23 | 19 |
| 30 | 47 | 53 | 60 | 53 |
| 31 | 53 | 52 | 57 | 59 |
| 32 | 42 | 48 | 41 | 46 |
| 33 | 35 | 49 | 78 | 82 |
| 35 | 18 | 20 | 9 | 9 |
| 37 | 25 | 36 | 41 | 56 |
| 38 | 2 | 9 | 6 | 8 |
| 39 | 79 | 78 | 46 | 44 |
| 41 | 3 | 13 | 18 | 12 |
| 42 | 68 | 73 | 63 | 65 |
| 43 | 10 | 15 | 24 | 33 |
| 44 | 62 | 77 | 60 | 66 |
| 45 | 10 | 16 | 8 | 10 |
| 46 | 52 | 53 | 73 | 51 |
| 47 | 22 | 69 | 26 | 38 |
| 48 | 30 | 66 | 24 | 37 |
| 49 | 52 | 60 | 8 | 6 |
| 50 | 31 | 37 | 12 | 12 |
| 51 | 26 | 34 | 70 | 70 |
| 52 | 30 | 63 | 68 | 76 |
| 53 | 72 | 79 | 67 | 66 |

## **Sanity check: Induction of cognitive load and fatigue via the TloadDback Task**

***Dual task Accuracy***

A weighted composite score was calculated across the total number of trials for each participant, as check for the attempted cognitive load while performing the TloadDback Task (see Smalle et al., 2021). Accuracy for letters (1-back letter detection; i.e., mean = 77 ± .13_SD_) and digits (parity number decision; .70 ± .25_SD_) represented 65% and 35% of the total score (Borragan et al., 2017). Performance of our participants can be seen in Figure 2. Most participants performed below the defined threshold of 85% accuracy (as defined during the pre-test), i.e., mean dual task accuracy = 75 ± .14*_SD_*, one-sample *t*_48_ = -5.09, *p* < .001, Cohen *d* = .7.

***Fatigue ratings***

As a manipulation check for the attempted induction of cognitive fatigue, we calculated summated rating scores for the items on the visual anologue subscale for fatigue that was presented pre and post the TloadDback task and the control task (see Figure 1). Overall, our participants reported higher feelings of cognitive fatigue as a result of performing the TloadDback task than as a result of performing the control task (i.e., Task x Time: *F*_1,46_ = 24.1, *p* < .001, *n^2^_p_* = .34). Post-hoc pairwise comparisons with Bonferroni correction showed higher levels of feeling of fatigue after the TloadDback task compared with the control task (i.e., post: paired-sample *t_46_ =* 2.19, *p* = .02, *d* = .32) while there were no differences in baseline ratings of fatigue (i.e. pre: *t* < 1). Likewise, the increase in fatigue was higher for the TloadDback task (i.e., pre versus post: paired-sample *t_48_ =* 4.5, *p* < .001, *d* = .64) than for the control task (i.e., pre versus post: *t* < 1). The fatigue rating scores are presented in Figure 2 (right panel).

##

**Figure S1. Cognitive Fatigue Induction**

**Table S3**. word structures per language (syllable streams), their Dutch pronunciation and summated bigram frequency

| Language | Syllable structures (orthography) | Syllable structures (IPA) | Summated bigram frequency |
| --- | --- | --- | --- |
| L1 | FI-MO-TI | /fi/-/mo/-/ti/ | 21655 |
|  | DA-MU-RI | /da/-/my/-/ri/ | 24556 |
|  | SA-RO-PU | /sa/-/ro/-/py/ | 27570 |
| L2 | PO-GI-LI | /po/-/ɡi/-/li/ | 23583 |
|  | BA-KO-RU | /ba/-/ko/-/ry/ | 24113 |
|  | GU-RA-KE | /ɣy/-/ra/-/kə/ | 27069 |
| L3 | SO-LE-VI | /so/-/lə/-/vi/ | 23172 |
|  | BE-TO-KU | /bə/-/to/-/kʏ/ | 23964 |
|  | HA-NO-SU | /ɦa/-/no/-/sy/ | 25777 |
| L4 | DE-WA-VO | /də/-/ʋɑ/-/vo/ | 23595 |
|  | KA-TA-KI | /ka/-/ta/-/ki/ | 24035 |
|  | BU-TE-FU | /by/-/tə/-/fy/ | 25516 |

**Table S4**. Individual performance on the executive function tasks. Norm scores in parentheses.

| **Participant** | **Forward DS** | **Backward DS** | **Switch cost (response time in ms)** | **Switch cost (errors)** | **Simon cost (response time in ms)** | **Simon cost (errors)** | **STD (in ms)** |
| --- | --- | --- | --- | --- | --- | --- | --- |
| 2 | 6 (97) | 5 (98) | 678 (92) | 1 (105) | 37 (94) | 6 (94) | 800 (103) |
| 3 | 6 (97) | 7 (114) | 231 (111) | 2 (99) | 20 (107) | 4 (101) | 1100 (75) |
| 4 | 8 (123) | 10 (139) | 728 (89) | 3 (93) | 11 (113) | 4 (101) | 900 (93) |
| 5 | 7 (110) | 5 (98) | 217 (111) | 0 (111) | 32 (97) | 0 (114) | 900 (93) |
| 6 | 7 (110) | 6 (106) | 264 (109) | 2 (99) | 48 (85) | 0 (114) | 700 (112) |
| 7 | 6 (97) | 6 (106) | 401 (104) | 0 (111) | 76 (63) | 17 (58) | 800 (103) |
| 8 | 4 (72) | 4 (90) | 473 (100) | 0 (111) | 39 (91) | 1 (111) | 800 (103) |
| 9 | 9 (135) | 6 (106) | 558 (97) | 1 (105) | 33 (96) | 0 (114) | 800 (103) |
| 10 | 4 (72) | 5 (98) | 2323 (20) | 7 (69) | 31 (98) | 0 (114) | 800 (103) |
| 11 | 9 (135) | 6 (106) | 578 (96) | 6 (75) | 31 (97) | 4 (101) | 1000 (84) |
| 12 | 6 (97) | 5 (98) | 91 (117) | 1 (105) | 28 (100) | 14 (68) | 900 (93) |
| 13 | 7 (110) |  | 731 (89) | 0 (111) | 5 (118) | -1 (117) | 1100 (75) |
| 14 | 6 (97) |  | 464 (101) | 0 (111) | 7 (116) | 8 (88) | 900 (93) |
| 15 | 6 (97) | 5 (98) | 236 (111) | 2 (99) | 38 (92) | 4 (101) | 700 (112) |
| 16 | 6 (97) | 5 (98) | 545 (97) | 0 (111) | 26 (102) | 14 (68) | 800 (103) |
| 17 | 7 (110) | 6 (106) | 650 (93) | 0 (111) | 34 (95) | 6 (94) | 700 (112) |
| 18 | 5 (84) | 5 (98) | 496 (99) | 2 (99) | 27 (101) | 2 (107) | 800 (103) |
| 19 | 6 (97) | 4 (90) | 713 (90) | 0 (111) | -3 (123) | 1 (111) | 800 (103) |
| 20 | 8 (123) | 6 (106) | 70 (118) | 1 (105) | 10 (114) | 1 (111) | 600 (122) |
| 21 | 6 (97) | 6 (106) | 167 (114) | 5 (81) | 18 (108) | 8 (88) | 800 (103) |
| 22 | 6 (97) | 6 (106) | 234 (111) | 7 (69) | 43 (89) | 8 (88) | 600 (122) |
| 23 | 4 (72) | 5 (98) | 421 (103) | 0 (111) | 50 (84) | 8 (88) | 600 (122) |
| 24 | 8 (123) | 3 (81) | 920 (81) | -3 (129) | 29 (99) | 0 (114) | 1200 (65) |
| 25 | 6 (97) | 6 (106) | 1016 (77) | 3 (93) | 28 (100) | 3 (104) | 1100 (75) |
| 26 | 6 (97) | 6 (106) | 645 (93) | -1 (117) | 10 (114) | -1 (117) | 900 (93) |
| 27 | 6 (97) | 7 (114) | 633 (93) | 1 (105) | -5 (125) | 4 (101) | 700 (112) |
| 28 | 7 (110) | 8 (122) | 525 (98) | 0 (111) | 33 (96) | 5 (97) | 700 (112) |
| 29 | 7 (110) | 7 (114) | 285 (109) | 0 (111) | 45 (87) | 10 (81) | 1200 (65) |
| 30 | 5 (84) | 4 (90) | 162 (114) | 2 (99) | 53 (81) | 11 (78) | 900 (93) |
| 31 | 5 (84) | 5 (98) | 297 (108) | 4 (87) | 17 (109) | 2 (107) | 600 (122) |
| 32 | 7 (110) | 8 (122) | 167 (114) | 1 (105) | 4 (119) | 0 (114) | 900 (93) |
| 33 | 7 (110) | 5 (98) | 339 (106) | 6 (75) | 14 (111) | 7 (91) | 800 (103) |
| 35 | 7 (110) | 7 (114) | 447 (102) | -1 (117) | 30 (99) | 5 (97) | 600 (122) |
| 37 | 6 (97) | 4 (90) | 453 (101) | 1 (105) | 25 (102) | 0 (114) | 700 (112) |
| 38 | 7 (110) | 8 (122) | 509 (99) | 0 (111) | 1 (121) | -2 (120) | 700 (112) |
| 39 | 6 (97) | 4 (90) | 295 (108) | 2 (99) | 52 (81) | 9 (84) | 500 (131) |
| 41 | 5 (84) | 7 (114) | 186 (113) | 0 (111) | 64 (72) | 3 (104) | 1000 (84) |
| 42 | 6 (97) | 3 (81) | 350 (106) | 3 (93) | 36 (94) | 6 (94) | 900 (93) |
| 43 | 7 (110) | 5 (98) | 601 (95) | -1 (117) | 17 (108) | 14 (68) | 800 (103) |
| 44 | 5 (84) | 7 (114) | 186 (113) | 3 (93) | -1 (122) | 6 (94) | 700 (112) |
| 45 | 5 (84) | 6 (106) | 269 (109) | 2 (99) | 5 (118) | 0 (114) | 800 (103) |
| 46 | 7 (110) | 5 (98) | 500 (99) | 4 (87) | 27 (101) | 5 (97) | 1100 (75) |
| 47 | 5 (84) | 3 (81) | 241 (110) | 2 (99) | 33 (96) | 5 (97) | 1000 (84) |
| 48 | 5 (84) | 6 (106) | 665 (92) | 7 (69) | 24 (103) | 3 (104) | 800 (103) |
| 49 | 8 (123) | 6 (106) | 449 (101) | 5 (81) |  | 0 (114) | 800 (103) |
| 50 | 5 (84) | 3 (81) | 223 (111) | 1 (105) | 30 (99) | -1 (117) | 800 (103) |
| 51 | 6 (97) | 4 (90) | 673 (92) | 8 (63) | 48 (85) | -1 (117) | 800 (103) |
| 52 | 5 (84) | 4 (90) | 769 (88) | 0 (111) | 83 (58) | 0 (114) | 900 (93) |
| 53 | 7 (110) | 4 (90) | 565 (96) | -1 (117) | 4 (119) | 4 (101) | 900 (93) |

| 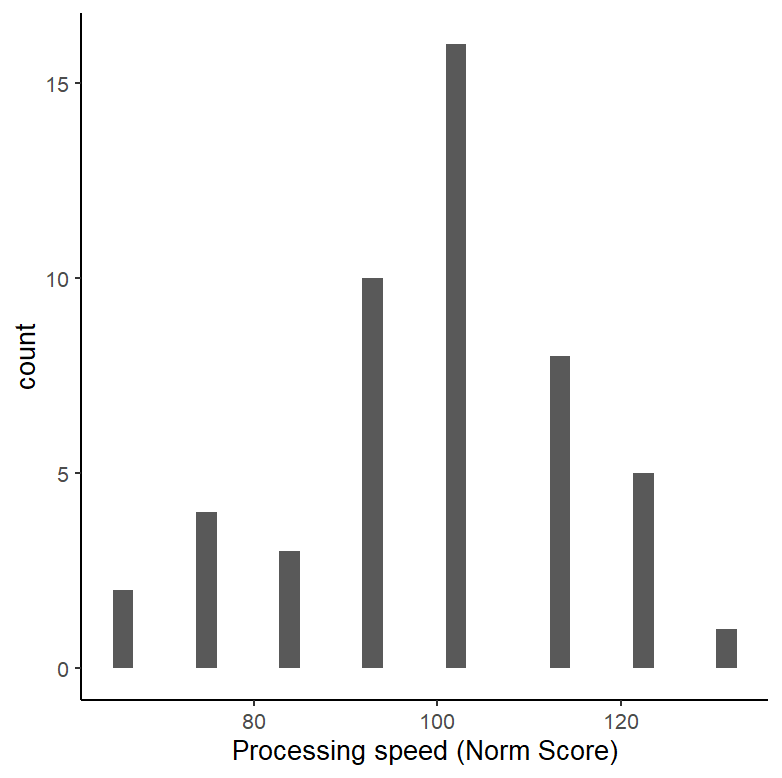 | 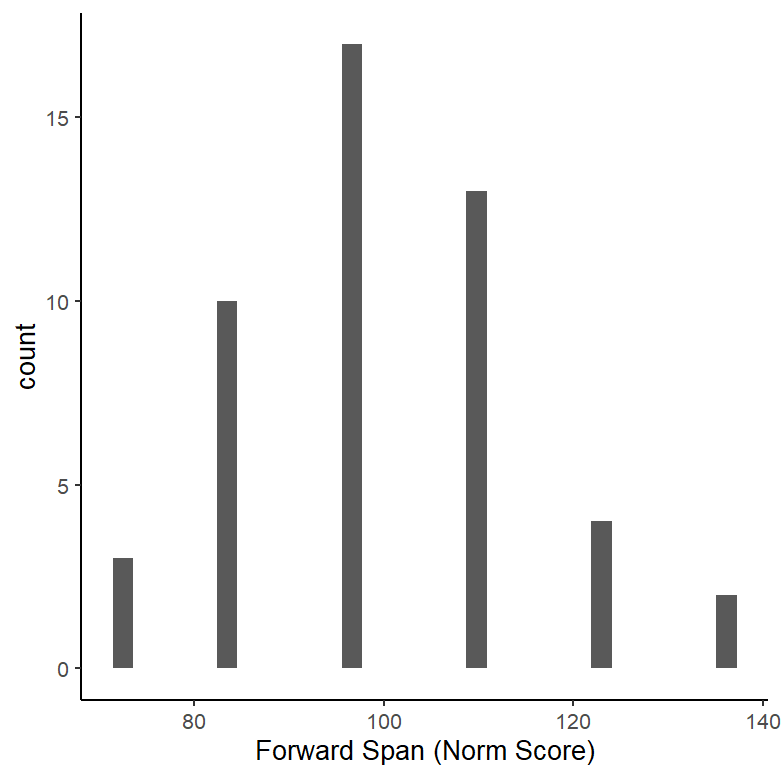 |
| --- | --- |
| 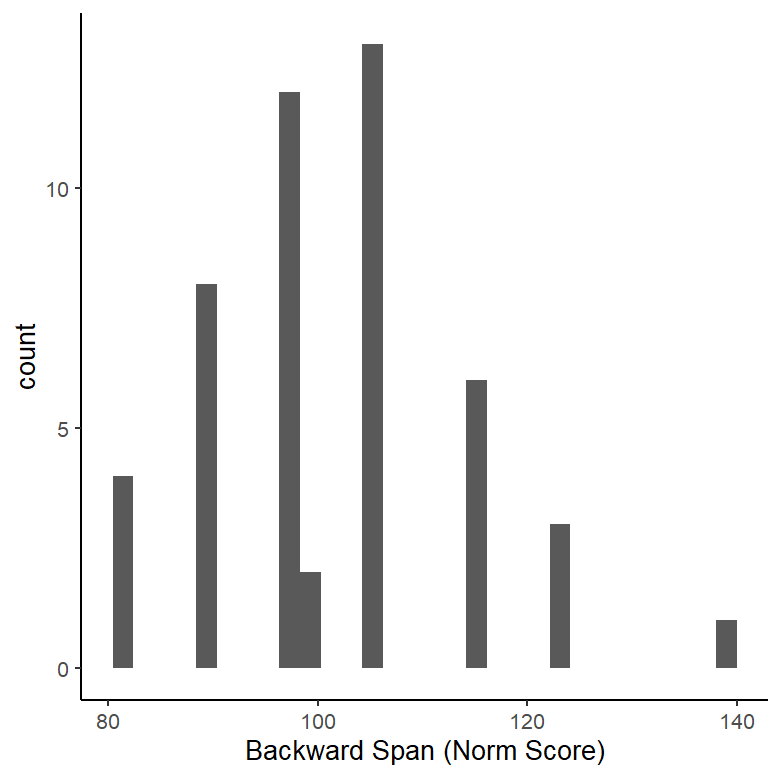 | 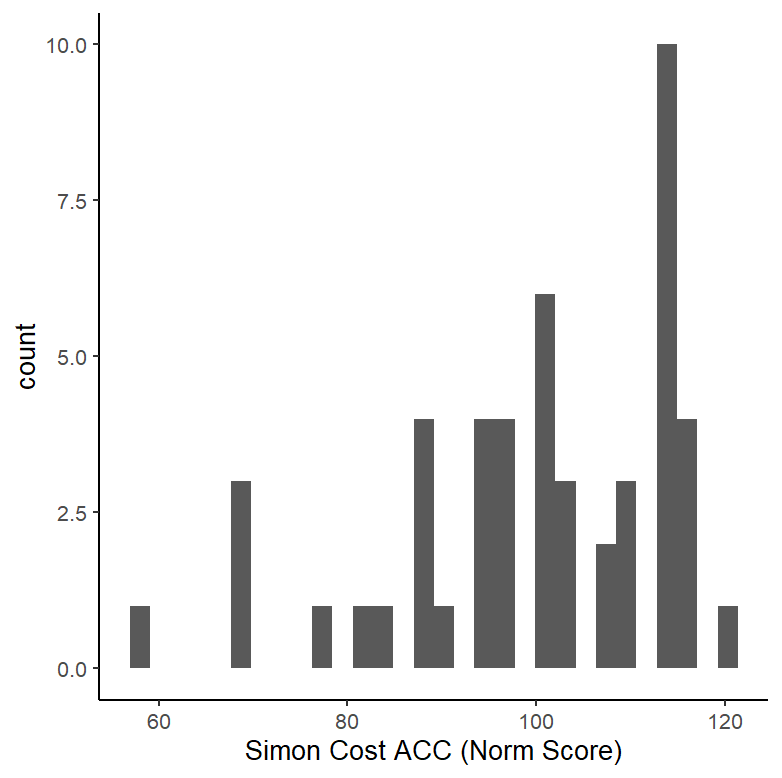 |
| 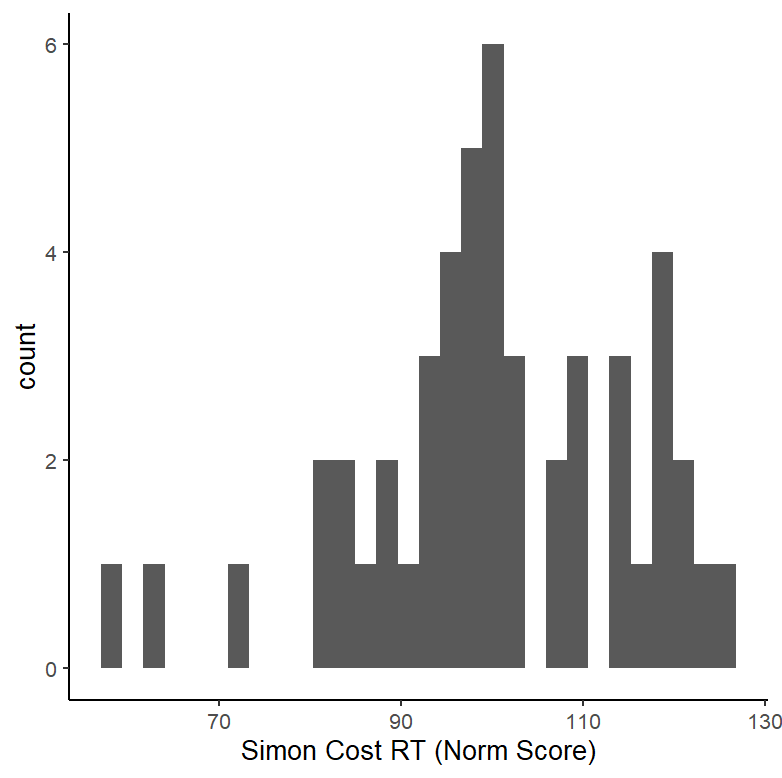 | 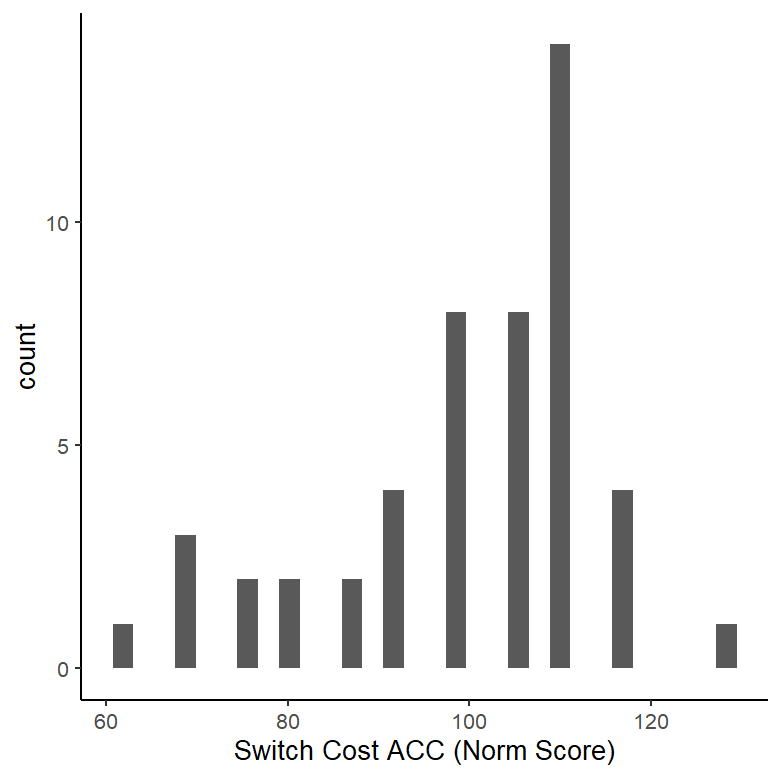 |
| 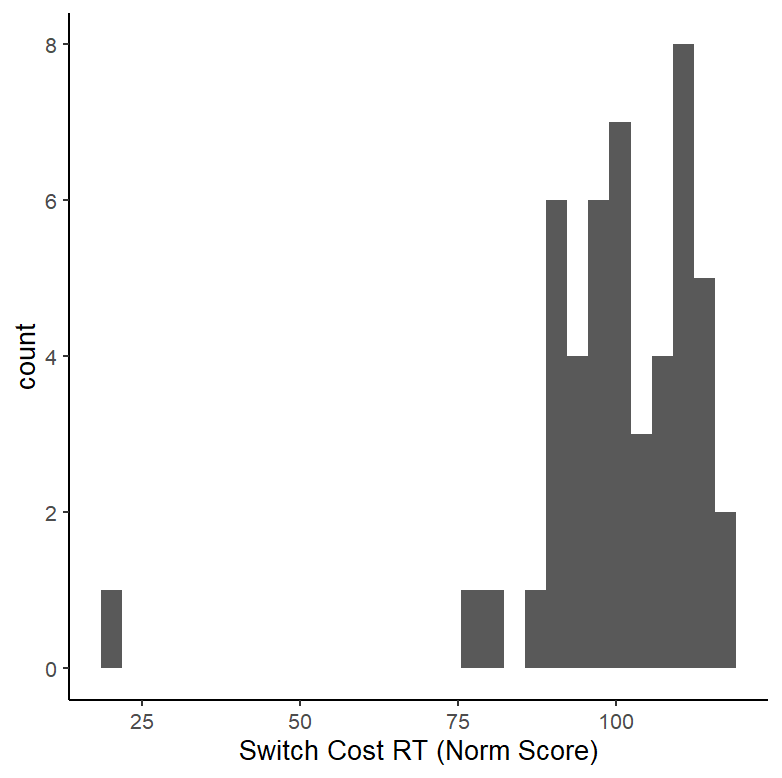 | 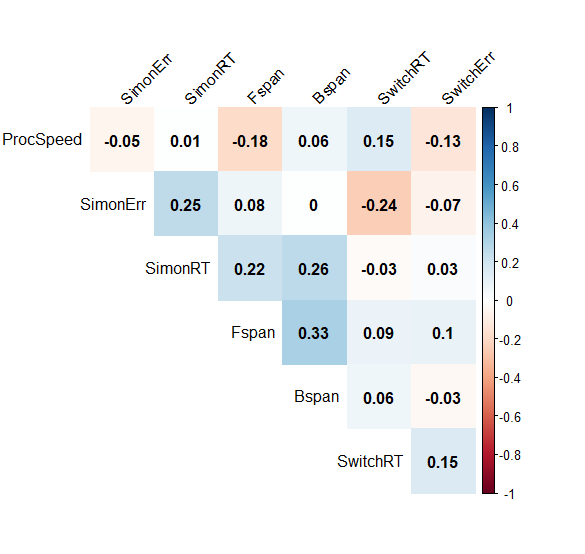 |

**Figure S2**. Histograms of the cognitive measures and their bivariate correlations

| 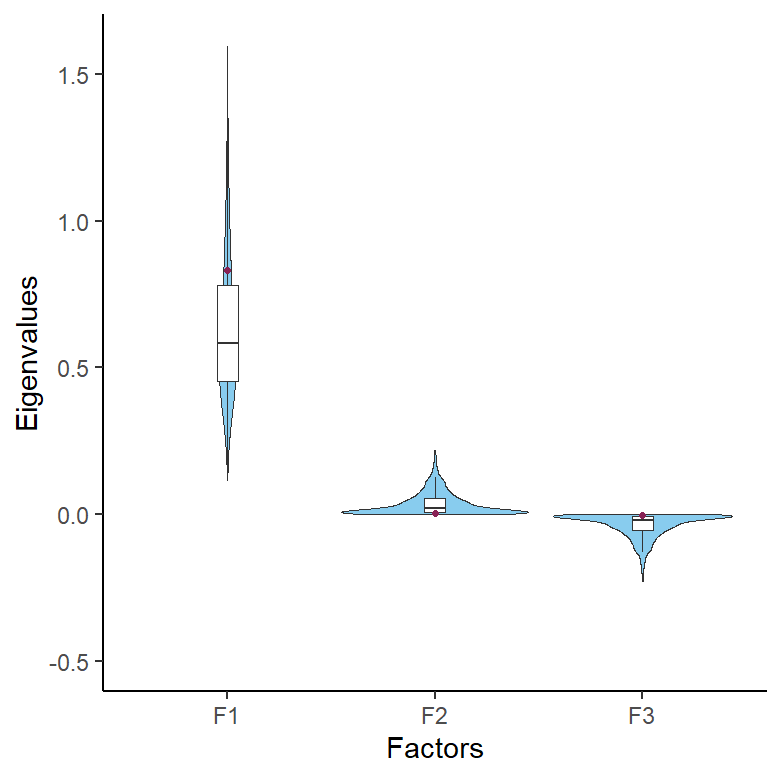 | 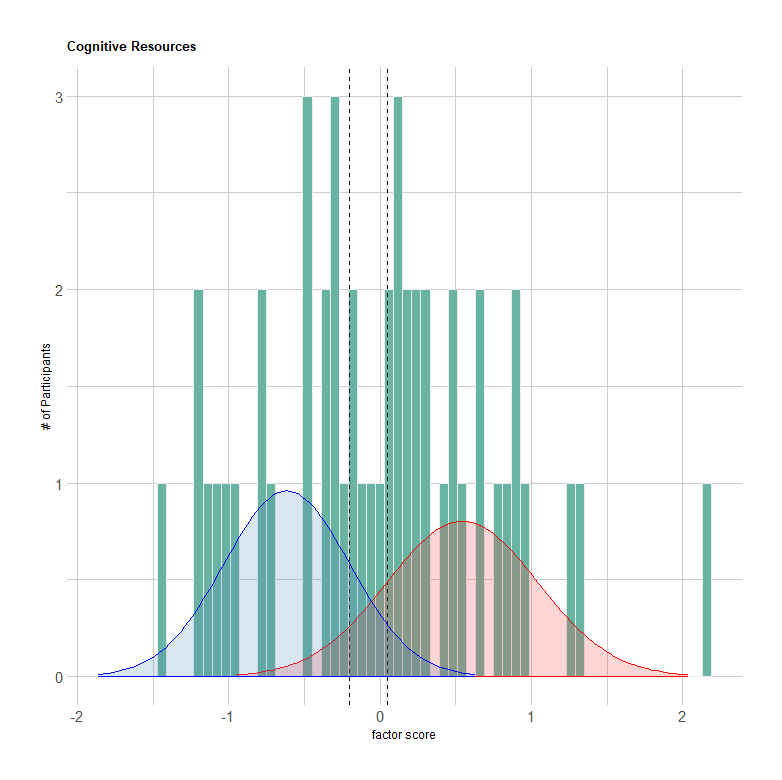 |
| --- | --- |

**Figure S3.** Parallel analysis to determine the extractable factors (left) and distribution of the extracted factor score with thresholds for defining the and low cognitive resource groups (right). Factor loadings are 0.417, 0.525, 0.624 for the Simon Cost, Forward and Backward Span, respectively.

**Table S4. Cognitive performance in the high and low cognitive resource group**

| **Cognitive measure** | **Cluster 1 (n = 19)** | **Cluster 2 (n = 23)** | **Independent t-test** |
| --- | --- | --- | --- |
| Processing Speed | M = 102.5, SD = 14.3 | M = 97.8, SD = 15.5 | ns |
| Simon Task (Δerrors) | M = 97.8, SD = 17.3 | M = 101.9, SD = 12.7 | *ns* |
| *Simon Task (Δresponse times) | M = 91.9, SD = 14.5 | M = 107.4, SD = 11.3 | *<.001* |
| *Forward Span | M = 90.5 SD = 11.4 | M = 108.4, SD = 12.6 | *<.001* |
| *Backward Span | M = 94.2, SD = 8.9 | M = 109.1, SD = 10.5 | *<.001* |
| Switch Task (Δerrors) | M = 98.5, SD = 16.9 | M = 101.3, SD = 13.3 | *ns* |
| Switch Task (Δresponse times) | M = 98.6, SD = 19.5 | M = 101.2, SD = 9.6 | *ns* |

*measures captured by the factor score
